# TongueBench: performance of prompt-engineered large multimodal models in Traditional Chinese Medicine tongue diagnosis

**DOI:** 10.3389/frai.2026.1943004

**Published:** 2026-10-21

**Authors:** Yihan Meng, Nuan Cui, Jingxin Yao, Shufeng Kong, Pei Liu, Zengbo Zhang, Hao Liang

**Affiliations:** 1 Institute of TCM Diagnostics, Hunan University of Chinese Medicine, Changsha, Hunan, China; 2 Provincial Key Laboratory of TCM Diagnostics, Hunan University of Chinese Medicine, Changsha, Hunan, China; 3 School of Software Engineering, Sun Yat-sen University, Zhuhai, China; 4 Department of Computer Science, Cornell University, Ithaca, NY, United States; 5 Academy of Chinese Medical Sciences, Hunan University of Chinese Medicine, Changsha, Hunan, China

**Keywords:** benchmarking, health informatics, knowledge-guided reasoning, large multimodal models, medical AI evaluation, prompt engineering, tongue diagnosis, Traditional Chinese Medicine

## Abstract

**Introduction:**

Traditional Chinese Medicine (TCM) tongue diagnosis requires reconstruction of multiple visual signs across tongue morphology, body color, coating texture, and coating color. We developed TongueBench as a prompt-controlled, ontology-constrained evaluation protocol for application programming interface (API)-accessed large multimodal models (LMMs), without task-specific training.

**Methods:**

Nine LMMs were evaluated on 2,694 tongue images with 22 expert-adjudicated labels under direct prompting (DP), knowledge-guided prompting (KGP), and knowledge-guided reflective prompting (KGRP). For the primary LMM-versus-supervised comparison, archived LMM predictions were matched to the same 270 test images used by three supervised baselines. Sensitivity analyses assessed a fixed label order, training-only frequencies, and no conflict removal. Raw-output auditing distinguished model-generated ontology violations from their removal by deterministic post-processing.

**Results:**

Across the nine evaluated models, KGRP increased mean Micro-F1 from 47.57% to 48.51%, Macro-F1 from 32.41% to 33.60%, and sample-level Jaccard from 34.45% to 35.32%, while reducing Hamming loss from 26.39% to 25.54%. Model-level changes were heterogeneous. Image-level bootstrap intervals described the fixed nine-model mean contrast under an unverified image-independence assumption. Supervised models had better observed aggregate scores on the matched split, but patient-level separation could not be established. Sensitivity analyses retained the direction of the mean KGRP-versus-DP changes.

**Discussion:**

TongueBench characterizes modest prompt-associated changes, parsing failures, repeated-output variation, and incomplete label-set reconstruction. These observations do not establish general prompt superiority, patient-level generalizability, or clinical utility.

## 1 Introduction

Tongue diagnosis is a component of Traditional Chinese Medicine (TCM) inspection and a structured visual assessment task in clinical practice. A clinician does not judge the tongue from one isolated sign. The diagnostic impression is assembled from tongue-body morphology, tongue-body color, fissures, tooth marks, coating thickness, coating texture, moisture, and coating color. These visual attributes are not independent: some labels are mutually exclusive, some commonly co-occur, and some differ only by subtle color or boundary cues. Tongue diagnosis is therefore better treated as ontology-constrained multi-label reasoning than as ordinary single-label image classification.

This property makes tongue diagnosis a demanding test case for large multimodal models (LMMs). A general-purpose LMM may correctly recognize several visible signs while still failing to recover the full expert label set. Conversely, a model may produce plausible text but violate the label ontology or omit diagnostically relevant coexisting features. Average accuracy alone is therefore insufficient. A trustworthy health informatics benchmark should assess whether a model maps an image to a fixed diagnostic ontology, whether repeated generations are stable, how prompt construction changes its output, and how zero-shot LMM behavior compares contextually with task-specific supervised references. Most recent progress in automated tongue-image analysis has come from supervised learning. Deep convolutional and multi-label models have been used to distinguish tongue lesions from normal images and recognize multiple tongue-image features in TCM diagnosis (Tiryaki et al., 2024; Liu et al., 2024a; Bhatnagar and Bansod, 2024). Related work has also improved dataset construction, image quality assessment, tongue segmentation, and end-to-end automated tongue-analysis workflows (Chen et al., 2025; Xian et al., 2022; Jin et al., 2025). These studies show that supervised or task-adapted models can learn useful tongue-image representations when annotated training data and model-specific optimization are available. They do not answer a different but increasingly relevant evaluation question: to what extent can API-accessed general-purpose LMMs perform this task when no task-specific training is performed and prompt construction is the main controllable design variable?

This question matters in health informatics because the observed performance of API-accessed medical foundation models depends not only on model parameters but also on task instructions, injected domain definitions, output schemas, and verification prompts. Prompt construction must therefore be treated as part of the evaluation protocol rather than as an incidental implementation detail (Schulhoff et al., 2024). InstructBLIP demonstrates multimodal instruction tuning across vision-language tasks (Dai et al., 2023), whereas prompt formulation can alter observed performance in language-model evaluations (Schulhoff et al., 2024; Sivarajkumar et al., 2024). Medical LMM studies further show that strong headline performance can coexist with protocol dependence, hidden reasoning flaws, and robustness limitations (Jin et al., 2024; Kaczmarczyk et al., 2024; Tanno et al., 2025). In TCM tongue diagnosis, where labels are definition-heavy and ontology-constrained, these risks may be particularly relevant.

TongueBench was designed to study prompt construction as a measurable inference-time intervention. We evaluated nine API-accessed LMMs on 2,694 tongue images, each annotated with a fixed 22-label TCM ontology, and compared three prompt strategies: direct prompting (DP), knowledge-guided prompting (KGP), and knowledge-guided reflective prompting (KGRP). The study asks four questions. First, does explicit TCM knowledge improve ontology-consistent zero-shot label reconstruction? Second, does reflective contradiction checking improve performance or change repeated-generation stability? Third, are prompt effects uniform across models and labels? Fourth, how should prompt-conditioned LMM performance be interpreted in the context of task-specific supervised reference models? The source code is publicly available at https://github.com/mengyiha/TongueBench-Open.

The contributions of TongueBench are four-fold. First, to our knowledge, TongueBench is the first large multimodal model benchmark for ontology-constrained TCM tongue diagnosis, using 2,694 images and 22 expert-adjudicated labels. Second, it evaluates prompt construction as a controlled inference-time intervention by comparing direct prompting, structured medical knowledge injection, and knowledge-guided reflective prompting under an otherwise fixed protocol. Third, it characterizes observable model behavior beyond aggregate performance through ontology-domain analysis, label-level prompt effects, repeated-generation stability, prompt sensitivity, and exact-match reconstruction. Fourth, it supports auditable medical AI assessment by linking model outputs and failure patterns to ontology-defined labels, mutual-exclusion rules, and a prespecified diagnostic ontology. These contributions position TongueBench as an evaluation protocol rather than a new diagnostic model or a claim of clinical deployment.

## 2 Related work

Automated tongue diagnosis has moved from single-feature recognition toward multi-label and structured modeling. Tiryaki et al. (2024) demonstrated deep-learning classification of tongue lesions and normal tongue images. Liu et al. (2024a)) extended tongue-image analysis to multi-label recognition of tongue features in stroke patients. Other recent systems have explored multi-label disease detection, multimodal tongue diagnosis, and multi-task segmentation-classification frameworks (Yang et al., 2025; Sha et al., 2025). TCM-specific visual-language modeling has also begun to appear in tongue-image description and constitution reasoning (Peng et al., 2026). The two architecture families used as supervised references in this study, Faster R-CNN and IF-RCNet, also provide established anchors for object-detection-based vision models and tongue-shape classification (Ren et al., 2015; Liang et al., 2025). Dedicated infrastructure studies have further addressed image quality control, standardized dataset construction, and end-to-end automated tongue-analysis workflows (Chen et al., 2025; Xian et al., 2022). These studies establish automated tongue diagnosis as an active medical AI research task, but they primarily focus on supervised learning, architecture design, or task-specific adaptation.

A second line of work concerns instruction-following LMMs. InstructBLIP demonstrates how instruction tuning can support generalization across vision-language tasks (Dai et al., 2023). This makes zero-shot medical image analysis technically plausible while establishing prompt construction and output control as explicit inference-time factors in multimodal model evaluation. A third line of work shows that prompt formulation can be an experimental variable rather than a cosmetic implementation choice. Promptception reported substantial prompt sensitivity in large multimodal models (Ismithdeen et al., 2025). Clinical prompting studies and medical question-answering benchmarks have similarly shown that prompt design can change measured model performance (Sivarajkumar et al., 2024; Maharjan et al., 2024). Multi-prompt evaluation and zero-shot benchmark construction further reinforce the need to report prompt design and evaluation protocol explicitly rather than treating them as implementation details (Polo et al., 2024; Pombal et al., 2025). Recent Chinese medical and TCM-specific language-model benchmarks make the same point for medical AI evaluation, where task design, safety checks, and domain reasoning must be reported as part of the benchmark rather than as *post hoc* narrative (Liu et al., 2024b; Kong et al., 2025). In-context multitask learning for TCM tongue diagnosis further supports the view that instruction design and task framing are substantive modeling choices rather than neutral wrappers (Fu et al., 2025). For tongue diagnosis, this is not a minor issue: labels such as pale tongue, light-red tongue, white coating, yellow coating, moist coating, and dry coating require both visual discrimination and correct use of domain definitions.

Static knowledge injection places fixed definitions in the prompt before inference. Retrieval-augmented generation (RAG) retrieves external documents at inference time and can provide source-linked or refreshed context. TongueBench implements only the former: KGP and KGRP use fixed ontology definitions and do not perform document retrieval, source attribution, or retrieval-freshness testing.

Finally, supervised baselines remain essential for interpreting zero-shot LMM results. A zero-shot LMM may be attractive because it requires no retraining, but its performance should still be interpreted against task-specific reference models developed for the corresponding diagnostic task (Kong et al., 2026). In this study, three supervised models serve as reference points. These baselines provide a task-specific reference for interpreting aggregate performance and exploratory label-level differences under the same image-level split.

## 3 Materials and methods

### 3.1 Study design

TongueBench was designed as a zero-shot multi-label benchmark in which prompt construction was the main inference-time intervention. All nine API-accessed LMMs were evaluated on the same image set, under the same 22-label ontology and output-normalization protocol. Three generations were requested per image and model-prompt condition as a resource-bounded probe of repeated-output variation. The retained number of generation records could be smaller and is reported separately from parsing success. Primary task-performance metrics used strict-majority voting over retained attempt vectors; attempt-level outputs supported the stability analysis. The archived scoring protocol was retained for the main results, and a separate sensitivity analysis varied only the conflict-resolution policy. This design does not characterize the full stochastic distribution of hosted API outputs.

The benchmark workflow consisted of image-level LMM inference, structured response parsing, label normalization, ontology-rule handling, voted aggregation, metric calculation, prompt-sensitivity analysis, and baseline comparison. This design allowed prompt effects to be interpreted within a fixed ontology-defined diagnostic label space rather than as artifacts of changing datasets, labels, or scoring rules (Figure 1).

**Figure 1:**
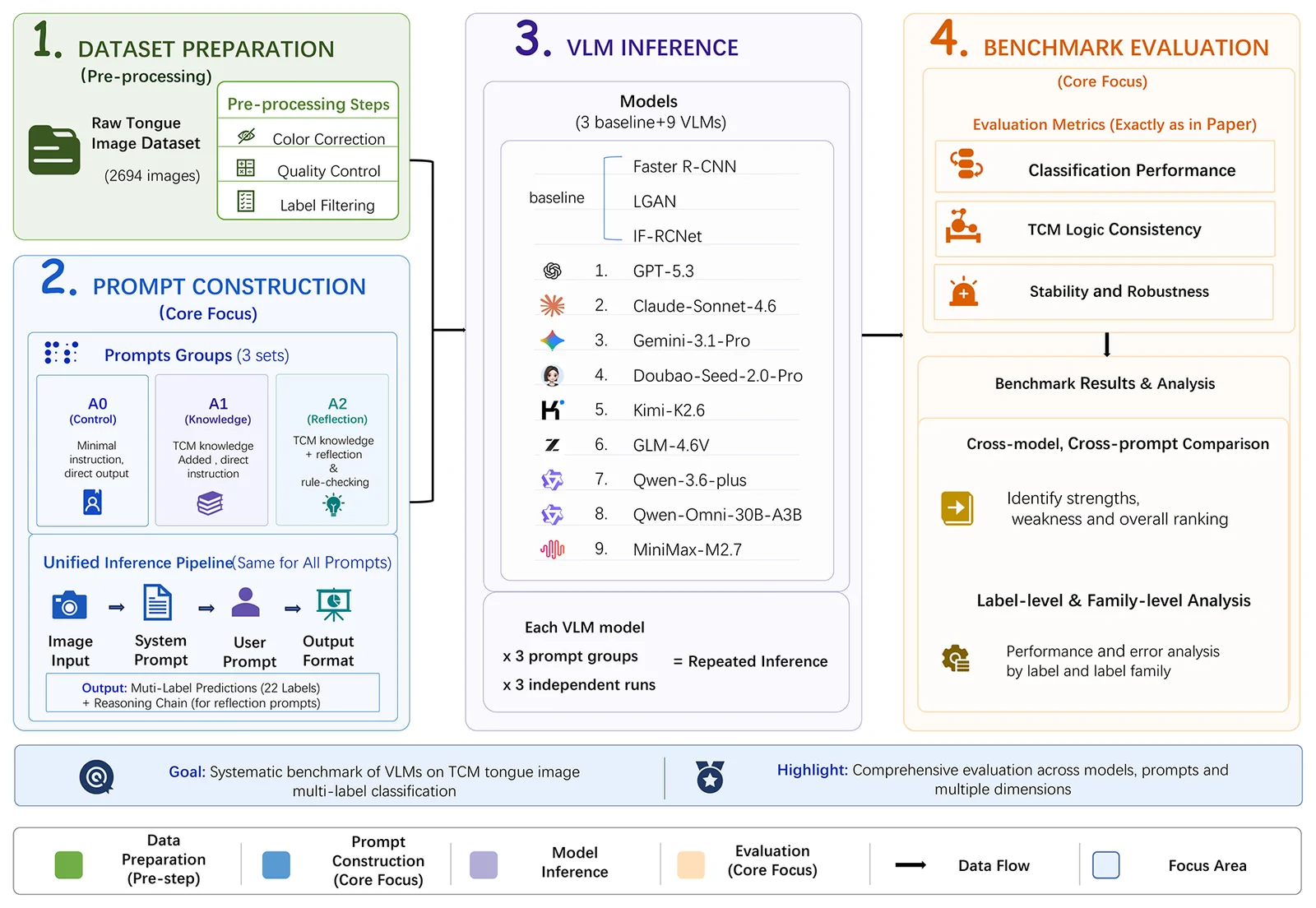
Analytical workflow of TongueBench. The benchmark proceeds from image-level zero-shot inference to structured label normalization, ontology-constrained conflict handling, voted aggregation, quantitative evaluation, and cross-model behavioral comparison.

### 3.2 Dataset and clinical ontology

According to the study protocol, the source cohort comprised tongue images from patients at the First Affiliated Hospital of Hunan University of Chinese Medicine and the Department of Traditional Chinese Medicine Oncology at the Fifth Affiliated Hospital of Sun Yat-sen University, together with healthy or subhealthy students at the Hanpu campus of Hunan University of Chinese Medicine. Images without a visible tongue, with incomplete or upward-curled tongue protrusion, or with excessive noise were excluded. Participants were generally seated (supine when severe pain required), supported by a chin rest; after 5–10 s of light stabilization, they opened the mouth and relaxed the tongue so that the tongue surface was flat and the tip naturally lowered. At least three images were acquired per participant at 15–20 s intervals. Retained images were denoised, illumination-normalized, and cropped to a square region approximately 1.5 times the tongue length, preserving part of the lips and surrounding skin.

The retained image-label files and split lists contain image identifiers but no validated participant-to-image linkage, patient counts, or per-participant retained-image counts. Filename components and acquisition descriptions are not a verified patient identifier. Consequently, repeated acquisitions cannot be reliably grouped by participant, and patient-level independence cannot be retrospectively established from these records. The image-level split described in Section 3.5 may therefore contain correlated images from the same participant across training, validation, and test subsets.

The reference standard was established through expert image annotation under the prespecified protocol. Three annotators with backgrounds in Chinese medicine reviewed each tongue image and assigned labels under a 22-label ontology. When two annotators agreed, or when an initial disagreement was resolved by discussion, the consensus label was accepted; unresolved disagreements were adjudicated by the third annotator. The supplied label workbook contains 2,695 unique image records; 2,694 had both valid images and reference annotations and were retained for analysis. All datasets are deposited on Zenodo and accessible via doi: 10.5281/zenodo.20701289.

Only the final consensus/adjudicated labels are available in the retained annotation archive; the original independent annotator labels and detailed adjudication records are unavailable. Inter-rater agreement, such as label-wise Cohen's kappa, cannot be calculated from the consensus labels. The reliability of the expert reference standard therefore remains unquantified, and no human performance ceiling is inferred.

The ontology contained four clinically interpretable domains. The tongue-body shape domain included tender tongue, tough tongue, enlarged tongue, thin tongue, tongue with spots or thorns, tongue with cracks, tongue with teeth marks, and ecchymosis. The tongue-body color domain included pale tongue, light-red tongue, dark-red and red tongue, and blue-purple tongue. The coating-texture domain included no tongue coating, thin tongue coating, thick tongue coating, moist tongue coating, dry tongue coating, rotten and greasy tongue coating, and peeled tongue coating. The coating-color domain included white, yellow, and gray-black tongue coating. The label “dark-red and red tongue” was used as a study-specific merged category corresponding to the traditional red-tongue and crimson-tongue concepts; this merge was fixed before evaluation and was not changed during *post hoc* analysis. The evaluator-side normalization used for the archived primary scores applied 12 pairwise mutual-exclusion rules: tender vs. tough tongue, enlarged vs. thin tongue, thin vs. thick coating, and no tongue coating vs. each of thin, thick, moist, dry, rotten and greasy, peeled, white, yellow, and gray-black coating. When both sides of a rule were present, the label with higher gold-standard support was retained, with a deterministic tie-break. The A2 model-facing prompt also described broader clinical checks for the tongue-color axis and moist-vs.-dry coating; those checks were treated as prompt instructions and were not additional evaluator-side rules in the archived scores. Blue-purple tongue was allowed to coexist with one primary color label or to appear alone. Label-support priorities were derived from the supplied reference-label workbook rather than from an independently specified clinical ordering or a training-only subset. The post-processed scores therefore characterize a pipeline that uses benchmark-level label-frequency information.

To assess dependence on benchmark-derived label priors, we additionally evaluated frequency-independent and training-frequency alternatives while holding the archived generation records and canonical scoring conventions fixed.

Figure 2 summarizes two dataset properties relevant to label-set reconstruction: unequal label frequencies and the frequent coexistence of multiple positive labels within an image. These properties motivate reporting both label-wise performance and complete label-set agreement.

**Figure 2:**
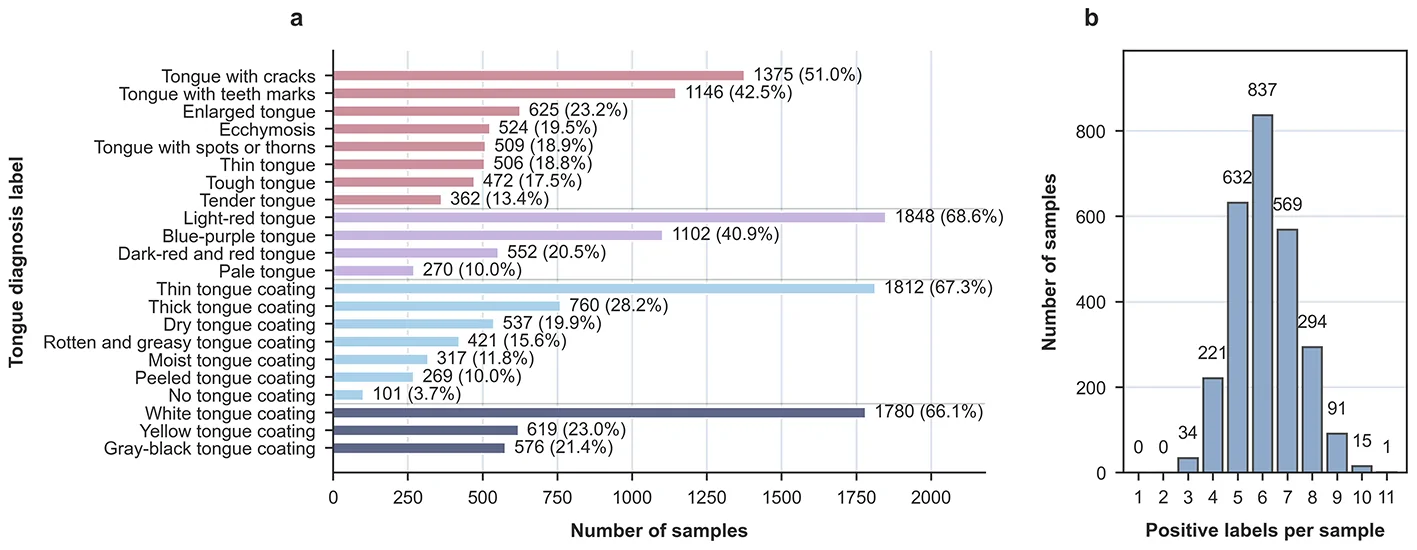
Joint structural overview of the TongueBench dataset. **(a)** shows sample support for each of the 22 clinical labels grouped by ontology domain, and **(b)** shows the distribution of positive-label cardinality per image. Analyses use the full 2,694-image benchmark.

### 3.3 Prompt construction strategies and evaluated LMMs

Archived model identifiers, endpoint hosts, access dates, image counts, and requested repeated-generation counts are reported in Supplementary Table S1. Temperature, top-p/top-k, maximum output tokens, seed, and retry behavior were not consistently recorded in the retained request archive. Their effective provider-side values, including any defaults, could not be independently verified or retrospectively reconstructed. Consequently, the historical API inference cannot be reproduced exactly, although the retained prompts, predictions, parsing rules, and local scoring procedures remain available for inspection and reanalysis.

Three prompt strategies were compared. DP provided the task instruction, the fixed 22-label inventory, and a structured output format. KGP retained the DP structure and added explicit TCM label definitions organized by the four ontology domains. KGRP extended KGP by adding contradiction review, mutual-exclusion checking, conservative rollback, and hard-case judgment before final label emission. These strategies formed a nested prompt ablation: task framing alone, task framing plus domain knowledge, and task framing plus domain knowledge plus rule-aware reflection. Across prompt conditions, the investigators kept the dataset, ontology, output schema, and scoring rules unchanged and performed no task-specific model training. Provider-side model updates could not be independently verified. This terminology also reflects that the systems were accessed through APIs rather than deployed locally.

### 3.4 Output normalization and behavioral evaluation

#### 3.4.1 Parsing and ontology-rule processing

Raw API responses were parsed into the required JSON schema. Label fields were normalized using the fixed alias lexicon, deduplicated, and filtered to the 22-label ontology. The archived post-processing pipeline applied the 12 evaluator-side mutual-exclusion rules sequentially to each attempt, retaining the label with greater reference-workbook support; ties used the lexical ordering of the canonical Chinese label strings. Canonical scoring then recomputed strict-majority votes from the processed attempt vectors. Raw and processed records were retained separately so that model-generated contradictions could be distinguished from their deterministic removal.

The raw-output audit and canonical scoring archive use distinct parsing records. In the raw audit, a parse failure is a retained generation that lacks an archived successful parse with a dictionary or list of labels. A valid empty output is an eligible parsed generation whose normalized label set is empty. A missing generation has no retained record for a requested slot. The canonical archive can classify some outputs differently, including list-shaped MiniMax-M2.7 responses, because of its processing conventions. We preserve each archive's own parsing flags and report the categories separately; an all-zero scoring vector does not reclassify a parse failure as a valid empty response.

For the additional raw-output audit, a generation was included only when its archived parse-success flag was true and its parsed output contained a dictionary or list of labels. Raw labels denote these outputs after alias normalization and ontology filtering but before mutual-exclusion removal. The same 12 rules were then applied to the same parsed generations. We report both the proportion of parsed generations containing at least one violation and the number of violated rules divided by 12 times the number of parsed generations. These denominators differ, so the generation-level conflict proportion is not E_single. Parse failures, valid empty label sets, and unrecorded generation slots are reported separately in Supplementary Table S12. This raw-output audit retains the original parsing flags and is separate from the canonical scoring records and the earlier permissive parser reconstruction.

Let *N* = 2,694 denote the number of images in the analysis, 2,694 for the full prompt benchmark and 270 for the supervised comparison. Let *L* = 22 denote the number of ontology labels. Three generations were requested per image and model-prompt condition, but the retained number of attempts, *K*_*i*_, could be smaller. For image (*i*), label (*j*), and retained attempt (*k*), (*yij*) denotes the expert reference label and ($\hat{y}ij^{\left(k\right)}$) denotes the normalized binary model prediction. The canonical scorer used a strict majority of the retained attempt vectors to obtain the voted prediction.

#### 3.4.2 Retained attempts and majority voting

Let *N* denote the number of evaluated images: 2,694 for the full prompt benchmark and 270 for the supervised comparison, and let *L* = 22. For image *i* in a model-prompt condition, *Gi* is the retained generation-record count (0–3), whereas *Ki* is the number of vectors supplied to canonical voting and stability scoring. Each retained generation supplies one vector, including an all zero vector when it has no usable labels. The archived scorer additionally reads a cached voted-label set when no generation is available. Three such records contained a cached empty set, giving *Gi* = 0 but *Ki* = 1; otherwise *Ki* = *Gi* in this archive. These cached vectors are not generated or successfully parsed responses. Let *yij* denote the reference label and *xij*(*k*) the label value in scored vector *k*. The voted prediction is defined in Equation 1 as


$$\begin{array}{l} {ỹ}_{ij}=\{\begin{array}{c} 1\left\{\overset{K_{i}}{\underset{k=1}{\sum }}x_{ij}^{\left(k\right)}>K_{i}/2\right\},K_{i}\geq 1,\\ 0,\;K_{i}=0. \end{array} \end{array}$$ (1)

The positive-vote thresholds are one when *Ki* = 1, two when *Ki* = 2, and two when *Ki* = 3. Ties are negative. Missing slots are not padded to three attempts. A recorded parse failure supplies a zero vector and remains in *Ki*; a valid empty output has the same numerical representation but a separate audit category. The three cached empty sets also yield negative votes and remain distinguishable from both categories. If no scored vector exists (*Ki* = 0), the voted vector is defined as all zero and the image remains in the task-performance denominator; this case did not occur after the archived cache-reading step. Supplementary Table S14 distinguishes generation counts, cached empty vectors, and scoring-vector counts.

This majority-vote rule is an aggregation step, not a performance metric. It was applied identically to all nine LMMs and all three prompt strategies before the primary accuracy metrics were calculated.

#### 3.4.3 Task performance and parsing success

The primary task-performance metrics were Micro-F1, Macro-F1, macro balanced accuracy, sample-level Jaccard, Hamming loss, exact-match ratio, and parse success rate. For Micro-F1, (TP), (FP), and (FN) denote true-positive, false-positive, and false-negative label decisions pooled over all images and labels after voting. Micro-precision measures how many predicted positive labels were correct, and micro-recall measures how many reference positive labels were recovered. Both quantities range from 0 to 1; higher values are better because they indicate fewer false positives or false negatives. Micro-precision and micro-recall were defined in Equation 2 as


$$\begin{array}{l} {\text{Precision}}_{\text{micro}}=\frac{\text{TP}}{\text{TP}+\text{FP}},\;{\text{Recall}}_{\text{micro}}=\frac{\text{TP}}{\text{TP}+\text{FN}}, \end{array}$$ (2)

Micro-F1 (Equations 3, 4) is the harmonic mean of micro-precision and micro-recall. It ranges from 0 to 1, and higher is better. In this dense multi-label task, Micro-F1 emphasizes the overall pool of label decisions and is therefore more influenced by common labels than Macro-F1.


$$\begin{array}{l} \text{Micro}-\text{F}1=\frac{2\cdot {\text{Precision}}_{\text{micro}}\cdot {\text{Recall}}_{\text{micro}}}{\text{Precisio}{\text{n}}_{\text{micro}}+{\text{Recall}}_{\text{micro}}}. \end{array}$$ (3)

For Macro-F1, (*F*1_*j*_) is the F1 score computed separately for label (*j*), and (*L* = 22) is the number of ontology labels. Macro-F1 is the arithmetic mean of the 22 label-wise F1 scores, so each label contributes equally regardless of prevalence.


$$\begin{array}{l} \text{Macro}-\text{F}1=\frac{1}{\text{L}}\overset{\text{L}}{\underset{\text{j}=1}{\sum }}{\text{F}1}_{\text{j}}. \end{array}$$ (4)

For balanced accuracy, (*Recallj*) is sensitivity for label (*j*), and (*Specificityj*) is the true-negative rate for the same label. Balanced accuracy ranges from 0 to 1, and higher is better. It is useful here because many tongue labels are imbalanced; averaging recall and specificity prevents a model from appearing strong merely because it predicts the majority negative state. Label-wise balanced accuracy is defined in Equation 5.


$$\begin{array}{l} \text{BalancedAccurac}{\text{y}}_{\text{j}}=\frac{\text{Recal}{\text{l}}_{\text{j}}+\text{Specificit}{\text{y}}_{\text{j}}}{2}, \end{array}$$ (5)

Macro balanced accuracy averages label-wise balanced accuracy across the ontology (Equation 6). It ranges from 0 to 1, and higher is better because it reflects stronger sensitivity-specificity balance across all labels.


$$\begin{array}{l} \text{Macro Balanced Accuracy}=\frac{1}{\text{L}}\overset{\text{L}}{\underset{\text{j}=1}{\sum }}\text{BalancedAccurac}{\text{y}}_{\text{j}}. \end{array}$$ (6)

For Hamming loss, *N* is the number of image count and *L* = 22 is the number of labels. The indicator equals 1 when the voted prediction differs from the reference for an image-label decision. Hamming loss ranges from 0 to 1; lower values indicate fewer incorrect label decisions. Hamming loss is defined in Equation 7.


$$\begin{array}{l} \text{HammingLoss}=\frac{1}{\text{NL}}\overset{\text{N}}{\underset{\text{i}=1}{\sum }}\overset{\text{L}}{\underset{\text{j}=1}{\sum }}1\left[{\text{y}}_{\text{ij}}\neq {\tilde{\text{y}}}_{\text{ij}}\right], \end{array}$$ (7)

For sample-level Jaccard, (*Y*_*i*_) is the set of reference-positive labels for image (*i*), and (${\tilde{\text{Y}}}_{\text{i}}$) is the set of voted positive labels. The numerator is the number of correctly overlapping labels, and the denominator is the union of all labels present in either the reference or prediction. Jaccard ranges from 0 to 1, and higher is better because it indicates stronger image-level overlap between the predicted and expert label sets. Image-level and sample-averaged Jaccard scores are defined in Equation 8.


$$\begin{array}{l} \text{Jaccar}{\text{d}}_{\text{i}}=\frac{\left|{\text{Y}}_{\text{i}}\cap {\tilde{\text{Y}}}_{\text{i}}\right|}{\left|{\text{Y}}_{\text{i}}\cup {\tilde{\text{Y}}}_{\text{i}}\right|},\text{ Jaccar}{\text{d}}_{\text{samples}}=\frac{1}{\text{N}}\overset{\text{N}}{\underset{\text{i}=1}{\sum }}\text{Jaccar}{\text{d}}_{\text{i}}, \end{array}$$ (8)

For exact-match ratio, (*Y*_*i*_) and $({\tilde{\text{Y}}}_{\text{i}}$) again denote the full reference and predicted label sets for image (*i*). The indicator equals 1 only when the complete multi-label phenotype is recovered without any missing or extra label. Exact-match ratio ranges from 0 to 1, and higher is better. It is intentionally stricter than F1 or Jaccard and should be interpreted as a complete-reconstruction stress test. The exact-match ratio is defined in Equation 9.


$$\begin{array}{l} \text{ExactMatch}=\frac{1}{\text{N}}\overset{\text{N}}{\underset{\text{i}=1}{\sum }}1\left[{\text{Y}}_{\text{i}}={\tilde{\text{Y}}}_{\text{i}}\right]. \end{array}$$ (9)

For parse success, the denominator is the number of retained generation records in the corresponding archive, *G* = Σ*iGi*, and the numerator is the number classified as successfully parsed by that archive. Neither missing slots nor cached empty scoring vectors count as generation records. Canonical parsing rates and raw-audit parsing counts must not be interchanged. Parse success measures output usability, not diagnostic accuracy. Parse success rate is defined in Equation 10.


$$\begin{array}{l} \text{ParseSuccessRate}=\frac{{\text{N}}_{\text{valid}}\text{generations}}{{\text{N}}_{\text{total}}\text{generations}}. \end{array}$$ (10)

#### 3.4.4 Repeated-generation stability and raw conflicts

Let *Ai*(*k*) denote the label set represented by scored vector *k*, *I*1 = *i*:*Ki*≥1, and *I*2 = *i*:*Ki*≥2. Pairwise Jaccard consistency first averages Jaccard similarity over all available pairs within each image in *I*2. Write *J*(*A, B*) = |*A*∩*B*|/|*A*∪*B*|, with $\text{J}\left(\text{A},\text{B}\right)=\{\begin{array}{c} \frac{\left|\text{A}\cap \text{B}\right|}{\left|\text{A}\cup \text{B}\right|},\text{A}\cup \text{B}\neq \emptyset ,\\ 1,\text{ A}=\text{B}=\emptyset . \end{array}\text{ J}\left(\emptyset ,\emptyset \right)=1$for this stability calculation. Thus, the image-level pairwise Jaccard score is defined in Equation 11 as


$$\begin{array}{l} \text{PairJaccar}{\text{d}}_{\text{i}}=\frac{2}{{\text{K}}_{\text{i}}\left({\text{K}}_{\text{i}}-1\right)}\overset{{\text{K}}_{\text{i}}-1}{\underset{\text{a}=1}{\sum }}\overset{{\text{K}}_{\text{i}}}{\underset{\text{b}=\text{a}+1}{\sum }}\text{J}\left({\text{A}}_{\text{i}}^{\left(\text{a}\right)},{\text{A}}_{\text{i}}^{\left(\text{b}\right)}\right),{\text{K}}_{\text{i}}\geq 2. \end{array}$$ (11)

Benchmark-level pairwise Jaccard consistency is the equal-weight mean over eligible images in *I*2, rather than over all *N* images (Equation 12). Larger values indicate greater similarity between repeated label sets.


$$\begin{array}{l} \text{Pairwise Jaccard Consistency}=\frac{1}{\left|{\text{I}}_{2}\right|}\underset{\text{i}\in {\text{I}}_{2}}{\sum }\text{PairJaccar}{\text{d}}_{\text{i}}. \end{array}$$ (12)

*Econsistent* measures the proportion of all available attempt pairs with exactly identical label sets. Unlike the image-averaged pairwise Jaccard statistic, this measure weights each available pair equally, as defined in Equation 13:


$$\begin{array}{l} {\text{E}}_{\text{consistent}}=\frac{\underset{\text{i}\in {\text{I}}_{2}}{\sum }\underset{1\leq \text{a}<\text{b}\leq {\text{K}}_{\text{i}}}{\sum }1\left\{{\text{A}}_{\text{i}}^{\left(\text{a}\right)}={\text{A}}_{\text{i}}^{\left(\text{b}\right)}\right\}}{\underset{\text{i}\in {\text{I}}_{2}}{\sum }\left(\frac{{\text{K}}_{\text{i}}}{2}\right)}. \end{array}$$ (13)

Images with fewer than two retained attempts contribute no pairs to E_consistent_ and are excluded from the pairwise Jaccard average. Two empty attempt sets receive a stability Jaccard value of 1; by contrast, the task-performance Jaccard convention assigns 0 to an empty reference–prediction union. Repeated empty or failed outputs can therefore increase apparent consistency without improving label reconstruction. The stored scorer returns zero when no eligible images or pairs exist; no such condition occurred in the 27 evaluated model-prompt combinations.

Let *R* = 12 denote the number of evaluator-side pairwise rules, and let *Vik* count the violated rules in scored vector *k* of image *i*. Canonical E_single_ divides the number of violations by the number of applicable scoring-vector rule checks are defined in Equation 14:


$$\begin{array}{l} {\text{E}}_{\text{single}}=\frac{\underset{\text{i}\in {\text{I}}_{1}}{\sum }\overset{{\text{K}}_{\text{i}}}{\underset{\text{k}=1}{\sum }}{\text{V}}_{\text{ik}}}{\text{R}\underset{\text{i}\in {\text{I}}_{1}}{\sum }{\text{K}}_{\text{i}}}. \end{array}$$ (14)

Canonical E_single_ includes all scored vectors, including zeros for unusable records and the three cached empty sets. Its post-processed zero value verifies deterministic conflict removal, not independent model compliance. For the separate raw audit, the analogous denominator is *R* times the number of eligible parsed generations; parse failures and cached empty sets are excluded. Generation-level raw conflict incidence instead counts eligible generations with at least one violated rule and divides by the number of eligible parsed generations.

For E_dynamic_, *Dir* equals 1 if the two labels in rule r occur in different scored vectors for image *i*, and 0 otherwise. Each image-rule combination is counted at most once. The canonical denominator includes all images with at least one scored vector (Equation 15):


$$\begin{array}{l} {\text{E}}_{\text{dynamic}}=\frac{\underset{\text{i}\in {\text{I}}_{1}}{\sum }\overset{\text{R}}{\underset{\text{r}=1}{\sum }}{\text{D}}_{\text{ir}}}{\text{R}|{\text{I}}_{1}|}. \end{array}$$ (15)

Images with *Ki* = 1 remain in the denominator of E_dynamic_ but contribute no cross-attempt drift event. This includes the three cached-empty cases with *Gi* = 0. Images with *Ki* = 0 would be excluded. This definition reproduces the archived scorer and differs from the *I*2 denominator used for pairwise Jaccard consistency. Lower E_dynamic_ indicates less cross-attempt switching between mutually exclusive labels.

#### 3.4.5 Prompt contrasts and uncertainty

Prompt sensitivity was further analyzed at two levels. For model (*m*) and label (*j*), (*Accuracymjp*) is the label-wise accuracy under prompt strategies (*p*), where (*p*∈*DP, KGP, KGRP*). (*VarAccmj*) is the variance of this accuracy across prompts (Equation 16). This is not an accuracy score; it is a prompt-sensitivity score. Lower values indicate more prompt-invariant behavior, whereas higher values indicate that the label decision is more strongly changed by prompt design.


$$\begin{array}{l} \text{VarAc}{\text{c}}_{\text{mj}}=\text{Va}{\text{r}}_{\text{p}\in \left\{\text{DP},\text{KGP},\text{KGRP}\right\}}\left(\text{Accurac}{\text{y}}_{\text{mjp}}\right). \end{array}$$ (16)

Model-level prompt sensitivity averages the label-level variance values over all (*L*) labels for model (*m*) (Equation 17). Lower values indicate greater prompt invariance at the model level, whereas higher values indicate stronger model-wide behavioral response to prompt changes.


$$\begin{array}{l} \text{PromptSensitivit}{\text{y}}_{\text{m}}=\frac{1}{\text{L}}\overset{\text{L}}{\underset{\text{j}=1}{\sum }}\text{VarAc}{\text{c}}_{\text{mj}}. \end{array}$$ (17)

For the aggregate prompt contrast, each expectation in Equation 18 denotes an equal-weight arithmetic mean across the nine fixed evaluated models. The contrast is therefore the mean within-model metric difference between KGRP and DP. Label-specific contrasts were computed separately for each label using the same model weighting.


$$\begin{array}{l} {\text{Δ}}_{\text{DP}\to \text{KGRP}}=\text{E}[{\text{Metric}}^{\text{KGRP}}]-\text{E}[{\text{Metric}}^{\text{DP}}]. \end{array}$$ (18)

The improvement direction depended on the metric. Positive shifts were favorable for Micro-F1, Macro-F1, macro balanced accuracy, sample-level Jaccard, exact-match ratio, parse success rate, pairwise Jaccard consistency, and *Econsistent*. Negative shifts were favorable for Hamming loss, *Esingle*, and *Edynamic*. This direction alignment was also used when summarizing composite prompt gains across Macro-F1, Micro-F1, sample-level Jaccard, and Hamming loss.

For prompt contrasts, the estimate was the equal-weight mean of the model-specific metric differences across the nine fixed, evaluated LMMs: one ninth of the sum of M(KGRP) minus M(DP), with KGP minus DP evaluated analogously. Using the archived canonical majority-voted predictions for 2,694 images, we performed 1,000 paired image-level bootstrap resamples with seed 20260823. Each resample used the identical image indices across all nine models and all three prompt strategies. Metrics were recomputed within each model and prompt before averaging the paired differences. Percentile intervals were calculated from the 2.5th and 97.5th percentiles. Model-specific KGRP-minus-DP intervals are reported alongside the fixed-model mean in Supplementary Table S11.

These intervals quantify sensitivity to image resampling conditional on the selected models, prompts, archived predictions, scoring rules, and voting procedure. They rely on an image-independence assumption that cannot be verified because participant identifiers are unavailable. Within-participant image correlation may make these intervals too narrow. A participant-cluster bootstrap could not be performed because validated participant identifiers were unavailable. The intervals do not estimate an effect in a hypothetical population of LMMs, uncertainty from new API calls or endpoint updates, or patient-level clinical generalizability. All interval-based interpretations are exploratory, without multiplicity-corrected superiority claims.

We also summarized five metrics across the nine evaluated models using their means, medians, and interquartile ranges. These metrics were Micro-F1, Macro-F1, sample-level Jaccard, Hamming loss, and exact-match ratio. Quartiles were calculated using linear interpolation. We also summarize the distribution of the nine paired KGRP-minus-DP model differences; this is distinct from subtracting the two prompt-wise medians. The analysis excluding MiniMax-M2.7 is retained as a sensitivity check, not as a replacement for the full nine-model results.

For reproducibility, the benchmark should be read together with the prompt files, normalization lexicon, post-processing scripts, and evaluation scripts. The three prompt IDs corresponded to DP, KGP, and KGRP; all required the same JSON fields and were processed by the same parser and ontology rules. This reporting choice is important because prompt effects in API-accessed LMMs cannot be interpreted without the exact prompt version, output schema, retry policy, aggregation rule, and metric direction.

Selected label-wise precision, recall, and F1 estimates in Supplementary Table S7 used the same image-resampling scheme and equal weighting across models. A prevalence-matched Bernoulli reference independently sampled each label according to its empirical prevalence. Its metric distribution over 1,000 replicates were summarized by the mean and central 95% simulation interval.

#### 3.4.6 Sensitivity to conflict-resolution priorities

We reanalyzed the retained generations under four evaluator policies: the archived benchmark-frequency rule; a fixed priority based on the existing 22-label ontology order; frequencies computed only from the 2,154 supervised training images; and no mutual-exclusion removal. The fixed order was selected without inspecting its scores and uses no reference-label frequencies. It is a deterministic sensitivity comparator, not a validated clinical priority. Rules were applied in the same sequential 12-pair order at attempt level. All policies retained the same image identifiers, canonical parsing flags, alias mapping, usable-label conventions, retained-attempt counts, strict-majority rule, and metric definitions. No new model inference or training was performed.

Before comparing policies, we reconstructed the benchmark-frequency pipeline and verified exact equality with all 27 archived canonical voted prediction matrices. We then recomputed Micro-F1, Macro-F1, sample-level Jaccard, Hamming loss, and exact-match ratio under each policy, reporting model-specific scores and equal-weight nine-model means on both the full 2,694-image set and the same 270 test images. Training-frequency priorities excluded validation and test images, but only the 270-image test analysis is held out from that frequency estimate; the full-set analysis includes the training images. Comparisons are descriptive sensitivity analyses without additional superiority tests. Supplementary Table S13 reports the priorities, aggregate scores, prompt contrasts, and prediction changes; the accompanying XLSX provides all model-specific results.

### 3.5 Supervised computer-vision baselines and API-accessed LMMs

The experimental comparison was structured around two different model families. The supervised baseline models were computer-vision (CV) systems trained on tongue images and task labels, so their performance reflects learned visual representations under a fixed train/validation/test split. The API-accessed LMMs were externally hosted multimodal foundation models queried in a zero-shot setting, so their performance reflects prompt-conditioned label reconstruction from images under a fixed ontology and output schema rather than task-specific weight optimization. This distinction is central to the experimental design: CV baselines test what supervised image models can learn from the benchmark, whereas API-accessed LMMs test what externally hosted multimodal systems can recover from instructions, domain definitions, and reflective constraints at inference time. We contextualized LMM performance using three representative supervised baselines: Faster R-CNN (Jiang et al., 2022), LGAN (Liu et al., 2024a), and IF-RCNet (Liang et al., 2025). Faster R-CNN used a ResNet-101 feature pyramid backbone adapted to image-level multi-label scoring, with tongue-mask-derived weak bounding boxes for detection targets (Ren et al., 2015). LGAN used convolutional feature extraction, label-guided attention, channel and spatial attention, and a bidirectional recurrent module for label-correlation modeling (Liu et al., 2024a). IF-RCNet is a two-level tongue segmentation and classification network based on mixed input and feature fusion; in this benchmark adaptation, it used RGB images plus tongue masks as mixed input and combined segmentation-derived features with classification features.

Archived baseline configuration files document image size 224, batch size 16, 200 maximum epochs, seed 42, no RandAugment, and early-stopping patience 20. Faster R-CNN used AdamW, learning rate 0.0003, mask-derived bounding-box targets, and ImageNet ResNet-101 weights in the reported pretrained configuration; LGAN used Adam with learning rate 0.001; IF-RCNet used Adam with learning rate 0.0001.

The supervised CV baselines used seed 42 and an image-level random 80/10/10 split of the 2,694-image benchmark, yielding 2,154 training images, 270 validation images, and 270 test images. The retained splitting code first selected 10% of image rows as test data and then one ninth of the remainder as validation data, without participant grouping. The selected Faster R-CNN, LGAN, and IF-RCNet split files contain the same 270 test-image identifiers. Because participant linkage is unavailable, the absence of participant overlap across subsets cannot be verified. LMM experiments did not train model weights on TongueBench; each model was queried under DP, KGP, and KGRP and evaluated using the same ontology and voting procedure.

The prompt-effects and repeated-generation analyses used all 2,694 evaluable images. The primary LMM-vs.-supervised comparison used only the same 270 test-image identifiers for both model families, as reported in Table 1, Figure 3, and Supplementary Table S2. Matching image identifiers resolves the sample-scope mismatch but does not establish patient-independent evaluation. Within-participant image correlation and possible participant overlap with supervised training data may make the supervised results optimistic. This single-split comparison is therefore descriptive and does not establish relative performance on independent patients.

**Table 1:** KGRP LMMs and supervised baselines evaluated on the same 270 test images.

Comparator	Micro-F1 (%)	Macro-F1 (%)	Sample-level F1 (%)	Hamming loss (%)	Sample-level Jaccard (%)
Doubao-Seed-2.0-Pro	59.11	43.85	59.04	24.55	44.10
Gemini-3.1-Pro	57.97	44.82	57.93	25.00	43.12
Claude-Sonnet-4.6	55.73	41.47	55.50	28.03	40.58
Kimi-K2.6	55.20	39.48	55.23	25.25	40.49
Qwen3.6-Plus	54.85	37.72	54.72	26.55	39.90
GPT-5.3	53.03	33.79	53.15	24.24	38.46
GLM-4.6V	50.23	27.58	50.65	24.12	36.49
Qwen3-Omni-30B-A3B	48.69	28.37	48.89	26.46	34.76
MiniMax-M2.7	0.48	0.28	0.28	27.86	0.18
Faster R-CNN	73.00	64.15	72.72	14.68	59.86
IF-RCNet	69.78	58.80	69.54	16.11	55.87
LGAN	68.63	58.17	68.61	16.46	54.72

Participant-level separation between the supervised training and test subsets could not be verified.

**Figure 3:**
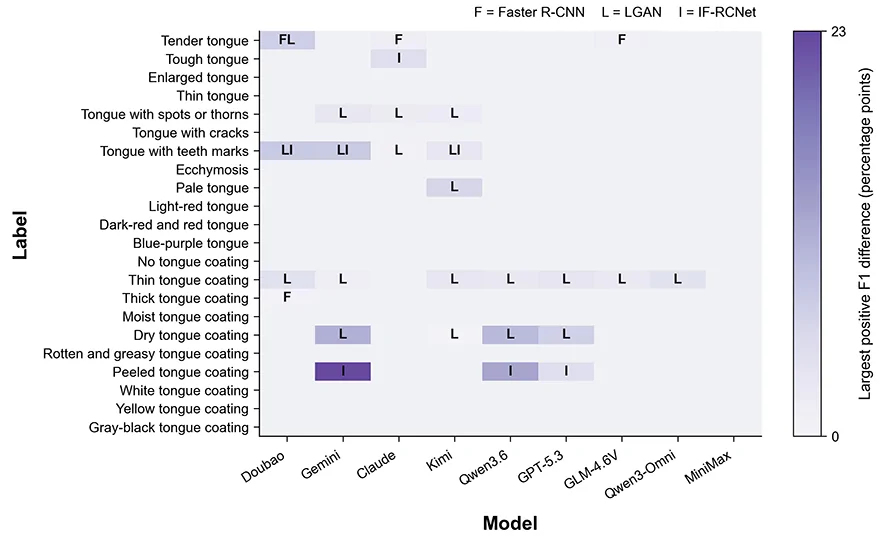
Exploratory label-level F1 differences between KGRP LMMs and supervised baselines on the same 270 test images. Rows represent the 22 labels, and columns represent the nine LMMs. Colored cells indicate higher observed KGRP F1 than at least one supervised baseline. F, L, and I denote Faster R-CNN, LGAN, and IF-RCNet, respectively. Color intensity represents the largest positive difference in percentage points for each model–label pair. These descriptive differences do not establish label-specific superiority, and patient-level separation of the image-based split remains unverified.

For the comparison in Table 1, sample-level F1 was reported as the mean image-level F1 across the same 270 test images; higher values indicate better per-image agreement. Analyses comparing LMM scores on all 2,694 images with supervised scores on 270 images were not used for the main comparative conclusions or Figure 3.

## 4 Results

### 4.1 Prompt construction strategy effects across LMMs and labels

Prompt-associated changes varied across the nine evaluated LMMs in Supplementary Table S3. KGRP yielded the highest Micro-F1 in seven models, whereas GPT-5.3 and Qwen3-Omni-30B-A3B performed best under DP. The strongest KGRP profiles were observed for Doubao-Seed-2.0-Pro and Gemini-3.1-Pro. Doubao-Seed-2.0-Pro reached Micro-F1 59.49%, Macro-F1 45.41%, and sample-level Jaccard 44.44%. Gemini-3.1-Pro reached Micro-F1 59.12%, Macro-F1 47.16%, macro balanced accuracy 66.29%, and sample-level Jaccard 44.04%. MiniMax-M2.7 was retained to show the full range of API-accessed model behavior, but it should be interpreted separately: even under KGRP, its Micro-F1 was only 0.36% and its parse success rate was 10.79%.

We next examined how prompt-associated changes varied across individual labels (Figure 4). Across model-prompt pairs, mean F1 was highest for white tongue coating (72.53%), thin tongue coating (71.69%), light-red tongue (68.31%), and tongue with cracks (62.61%). Lower values were observed for thin tongue (5.29%), no tongue coating (5.61%), blue-purple tongue (11.05%), gray-black tongue coating (11.46%), ecchymosis (12.72%), and pale tongue (13.79%). The largest mean DP-to-KGRP gains were tender tongue (+10.22 percentage points), no tongue coating (+5.15), tough tongue (+4.26), dark-red and red tongue (+4.22), blue-purple tongue (+3.93), thick tongue coating (+3.53), and thin tongue coating (+3.40). Tongue with teeth marks (−7.19), pale tongue (−4.51), gray-black tongue coating (−2.37), and ecchymosis (−2.29) declined. Prompt-associated changes were therefore heterogeneous across the ontology.

**Figure 4:**
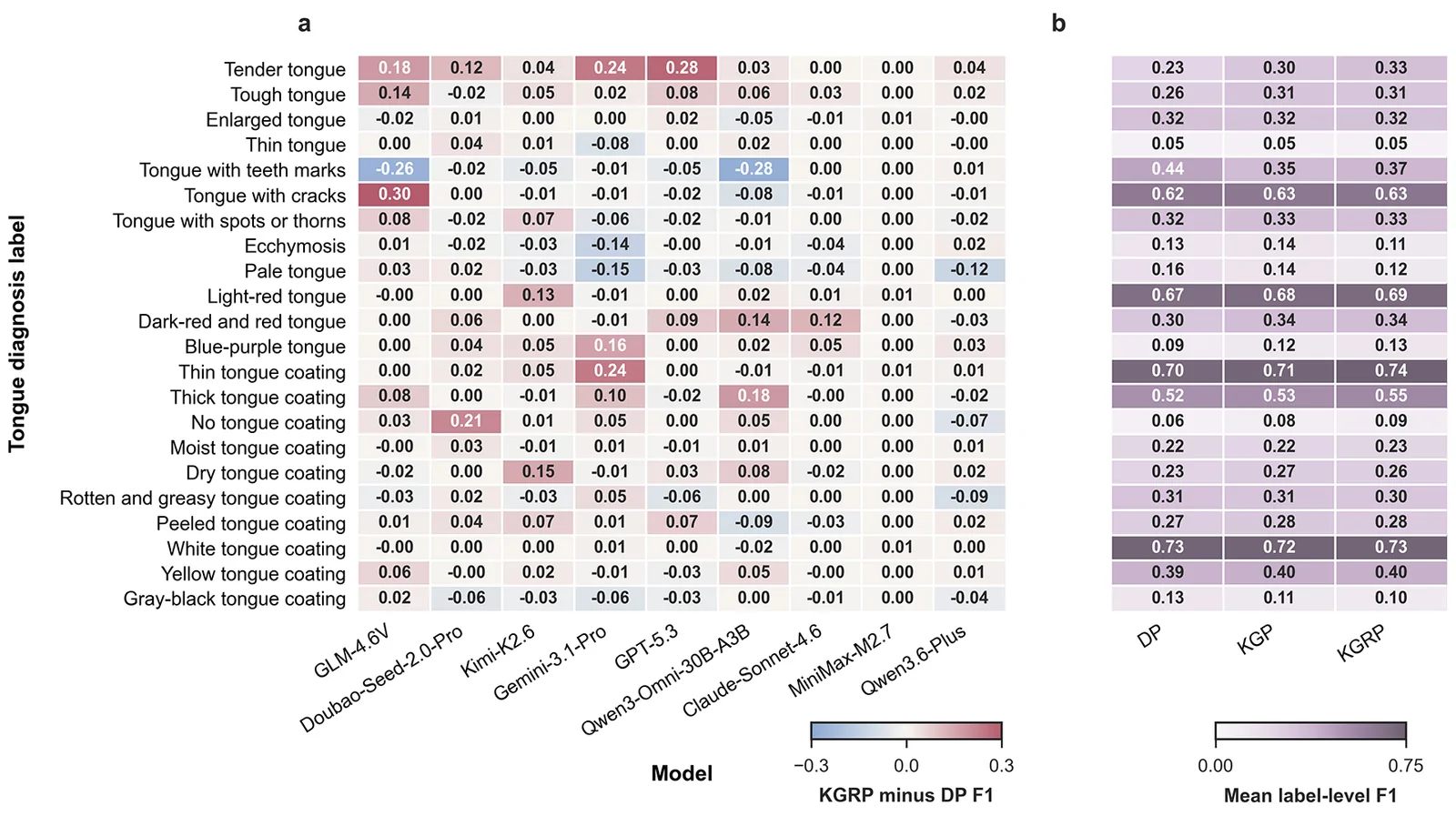
Dual-perspective summary of label behavior. **(a)** shows the model-specific F1 change from DP to KGRP for each label. **(b)** shows prompt-wise mean F1 for each label after averaging across models.

Figure 5 summarizes direction-aligned relative changes across four performance metrics for each model. Figure 5 further shows that KGRP's aggregate advantage was driven by a subset of responsive models. The largest DP-to-KGRP Micro-F1 increases were observed in Gemini-3.1-Pro (+3.65 percentage points), Kimi-K2.6 (+2.35 percentage points), Doubao-Seed-2.0-Pro (+2.09 percentage points), and GLM-4.6V (+1.32 percentage points). GPT-5.3 and Qwen3-Omni-30B-A3B moved in the opposite direction. Thus, the result is not a universal prompt-ranking claim; it is evidence that prompt construction strategy induces heterogeneous model-specific behavior.

**Figure 5:**
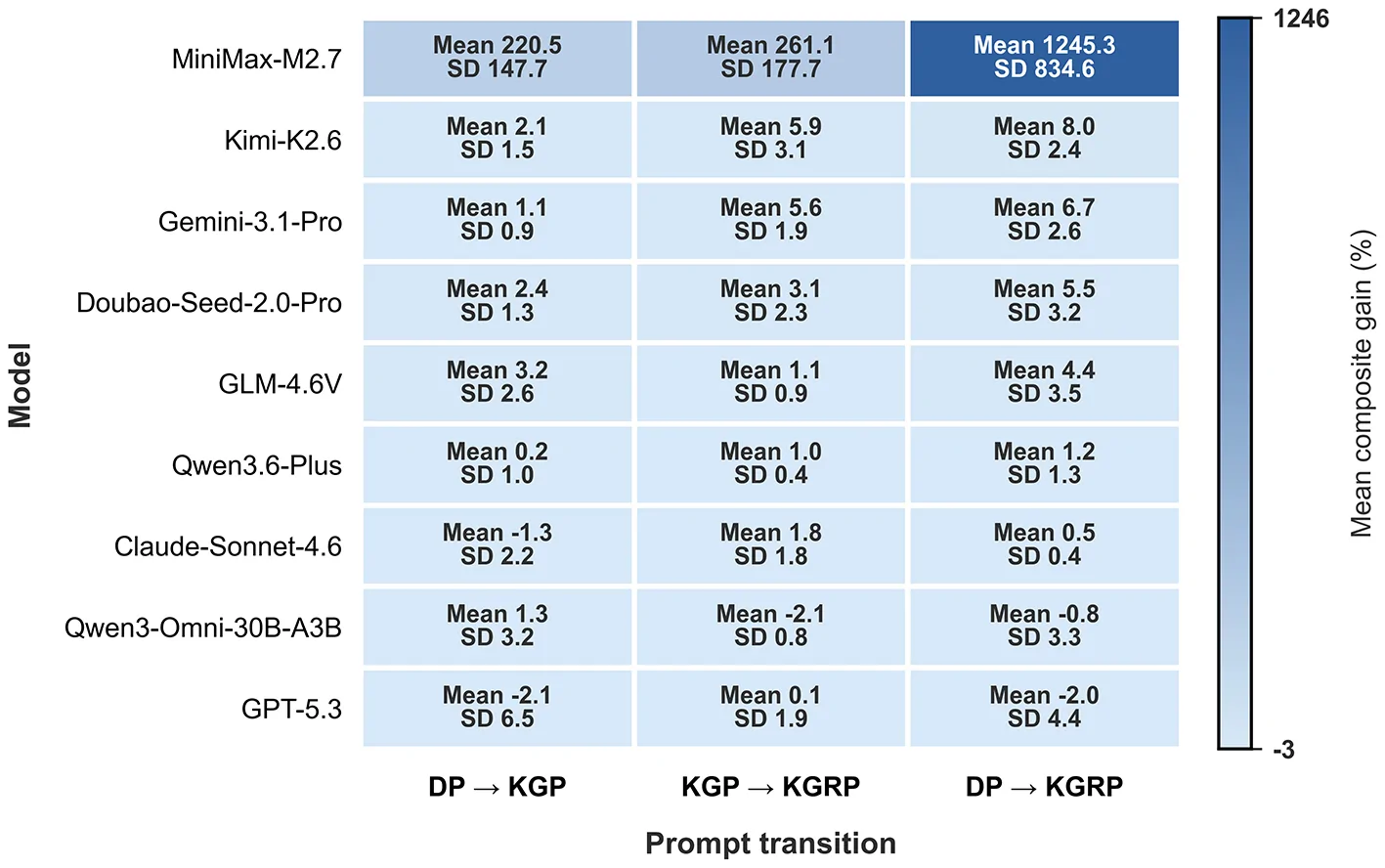
Model-level composite prompt gain across four key benchmark metrics for the transitions DP to KGP, KGP to KGRP, and DP to KGRP. Positive values indicate improvement after direction alignment across Macro-F1, Micro-F1, sample-level Jaccard and Hamming loss. Each cell summarizes the mean percentage gain and cross-metric standard deviation across the four metrics; larger values are favorable for Macro-F1, Micro-F1, and sample-level Jaccard, whereas smaller values are favorable for Hamming loss. The MiniMax-M2.7 model is retained to show the full range of failure and response behavior, but its extreme values should be interpreted separately from the remaining models.

Using the stored voted predictions, the model-level Prompt Sensitivity was: Doubao-Seed-2.0-Pro 0.001051; Gemini-3.1-Pro 0.001165; GPT-5.3 0.000936; Qwen3-Omni-30B-A3B 0.000789; Kimi-K2.6 0.000390; Qwen3.6-Plus 0.000295; GLM-4.6V 0.000271; Claude-Sonnet-4.6 0.000216; and MiniMax-M2.7 0.0000001. These are descriptive prompt-sensitivity summaries, not significance tests or evidence of clinical reliability.

### 4.2 Aggregate accuracy and ontology-domain performance

Table 2 summarizes the equal-weight mean performance of the nine evaluated LMMs under each prompt strategy. The direction of interpretation follows Section 3.4: higher Micro-F1, Macro-F1, macro balanced accuracy, sample-level Jaccard, exact-match ratio, and parse success rate are better, whereas lower Hamming loss is better. KGRP gave the strongest aggregate profile. Relative to DP, it increased mean Micro-F1 from 47.57% to 48.51%, Macro-F1 from 32.41% to 33.60%, macro balanced accuracy from 58.75% to 59.38%, and sample-level Jaccard from 34.45% to 35.32%. Hamming loss decreased from 26.39% to 25.54%. KGP showed smaller changes in the aggregate means, while KGRP yielded the highest mean F1 and Jaccard scores and the lowest mean Hamming loss. Exact-match ratio increased only from 0.40% to 0.49%, and parse success rate increased from 89.70% to 89.83%, so KGRP improved the aggregate profile without solving complete label-set reconstruction or structured-response reliability.

**Table 2:** Mean benchmark metrics across the nine evaluated LMMs for each prompt strategy.

Metric	DP (%)	KGP (%)	KGRP (%)
Mean micro-F1	47.57	47.68	**48.51**
Mean macro-F1	32.41	33.22	**33.60**
Mean macro balanced accuracy	58.75	59.18	**59.38**
Mean sample-level Jaccard	34.45	34.46	**35.32**
Mean Hamming loss	26.39	26.20	**25.54**
Mean exact-match ratio	0.40	0.40	**0.49**
Mean parse success rate	89.70	89.39	**89.83**

Median and interquartile-range summaries are reported in Supplementary Table S10.

Bold values indicate the best-performing results.

Under the exploratory image-level paired bootstrap, the fixed nine-model mean KGRP-minus-DP difference was +0.94 percentage points for Micro-F1 (95% percentile interval, +0.77 to +1.10), +1.19 points for Macro-F1 (+0.93 to +1.44), +0.87 points for sample-level Jaccard (+0.71 to +1.02), and −0.85 points for Hamming loss (−0.94 to −0.76). The exact-match interval included zero. For KGP minus DP, the Macro-F1 and Hamming-loss intervals were favorable, whereas the Micro-F1 and Jaccard intervals included zero. These intervals are conditional on the fixed benchmark configuration and may understate uncertainty from within-participant image correlation; they are not evidence of general superiority across LMMs.

Model-specific Micro-F1 differences ranged from −1.19 percentage points for Qwen3-Omni-30B-A3B to +3.65 points for Gemini-3.1-Pro. The corresponding exploratory intervals were −1.79 to −0.56 and +3.08 to +4.21 points. GPT-5.3 also decreased (−1.11 points; −1.53 to −0.66), whereas the Claude-Sonnet-4.6 interval included zero (+0.29 points; −0.12 to +0.68). Supplementary Table S11 reports the model-specific contrasts and makes clear that KGRP was not uniformly beneficial.

Across the nine evaluated models, the median Micro-F1 was 54.40% (IQR, 49.27%−55.38%) under DP, 53.52% (49.72%−54.84%) under KGP, and 55.18% (49.98%−55.67%) under KGRP. The median paired KGRP-minus-DP change was +0.69 percentage points (IQR, +0.29 to +2.09). These summaries describe variation among the nine evaluated models and do not support inference to a broader model population. Supplementary Table S10 reports the corresponding summaries for all five metrics.

Taken together, these directionally consistent changes indicate a modest aggregate gain under KGRP, although the model-specific trajectories in Figure 6 show that the gain was not uniform.

**Figure 6:**
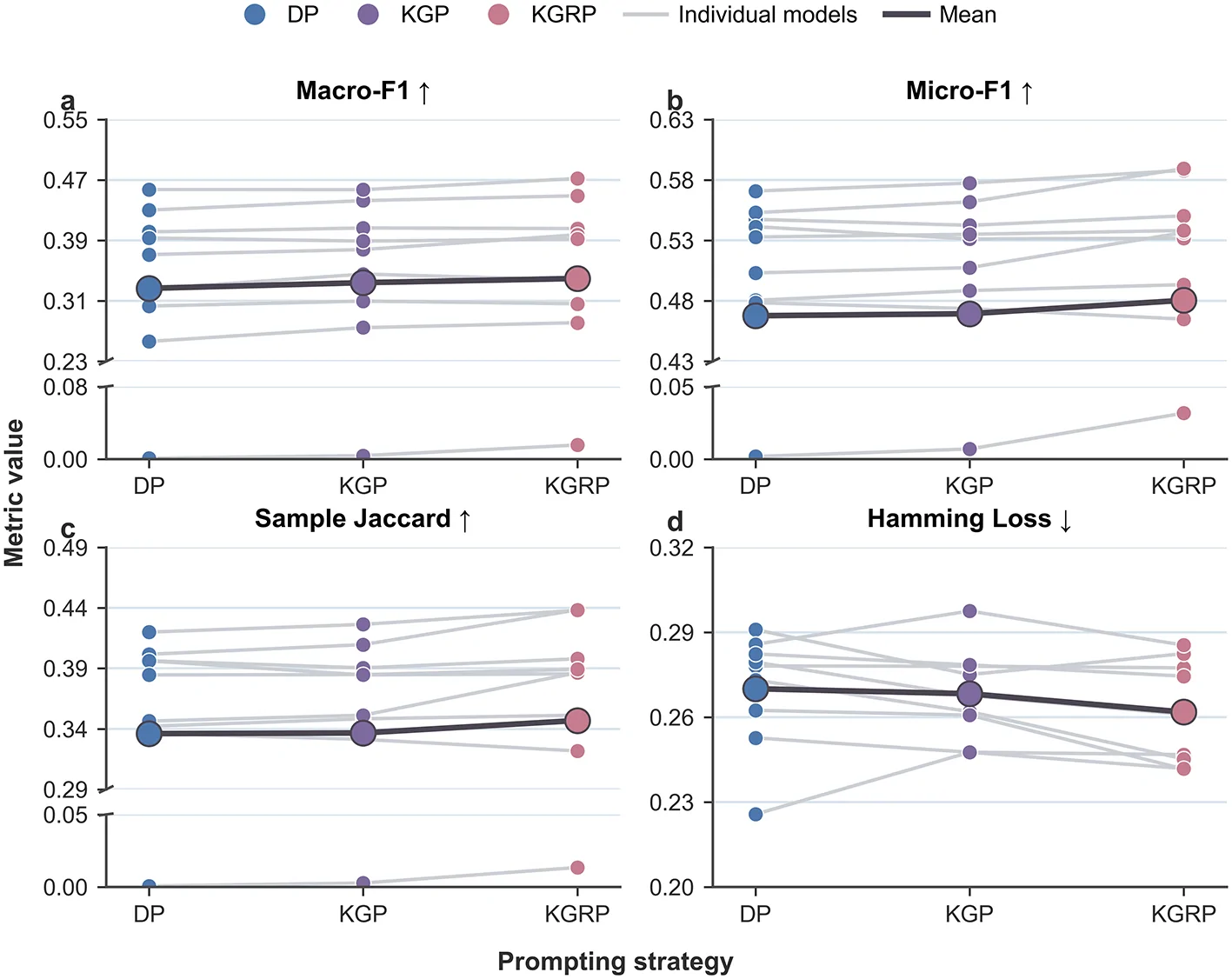
**(a–d)** show prompt-associated trajectories of Macro-F1, Micro-F1, sample-level Jaccard, and Hamming loss, across the nine evaluated models. Thin gray lines denote individual models and bold black lines denote prompt-wise means. Broken y-axes are used where needed to display the MiniMax-M2.7 outlier without compressing the remaining LMMs.

Domain-level analysis localized the aggregate changes mainly to tongue-body color and coating texture in Table 3. For tongue-body color, mean Micro-F1 increased from 49.35% under DP to 51.58% under KGRP, and sample-level Jaccard increased from 46.91% to 49.09%. For coating texture, mean Micro-F1 increased from 48.00% to 49.92%, Macro-F1 from 32.67% to 34.81%, and Hamming loss decreased from 23.10% to 21.91%. Tongue-body shape changed little in Micro-F1, and coating-color performance was broadly stable. These findings extend the label-level results in Figure 4 by showing where changes were concentrated within the ontology.

**Table 3:** Ontology-domain performance by prompt construction strategy.

Ontology domain	Metric (%)	DP (%)	KGP (%)	KGRP (%)
Tongue-body shape	Micro-F1	**38.77**	37.95	38.71
	Macro-F1	29.77	30.31	**30.60**
	Macro balanced accuracy	58.41	59.11	**59.28**
	Sample-level Jaccard	26.39	25.88	**26.50**
	Hamming loss	29.69	29.52	**28.88**
Tongue-body color	Micro-F1	49.35	50.81	**51.58**
	Macro-F1	30.53	**32.01**	31.94
	Macro balanced accuracy	56.03	**56.58**	56.57
	Sample-level Jaccard	46.91	48.24	**49.09**
	Hamming loss	27.24	26.51	**26.31**
Coating texture	Micro-F1	48.00	48.41	**49.92**
	Macro-F1	32.67	33.77	**34.81**
	Macro balanced accuracy	60.75	60.98	**61.45**
	Sample-level Jaccard	39.87	39.67	**41.32**
	Hamming loss	23.10	23.11	**21.91**
Coating color	Micro-F1	**60.70**	60.65	60.67
	Macro-F1	**41.31**	41.29	40.96
	Macro balanced accuracy	58.59	**58.66**	58.53
	Sample-level Jaccard	**57.80**	57.72	57.69
	Hamming loss	24.11	24.13	**24.04**

Values are model-averaged results across the nine API-accessed LMMs after majority voting. Metrics are reported separately for the four prespecified ontology domains and include Micro-F1, Macro-F1, macro balanced accuracy, sample-level Jaccard, and Hamming loss. Higher values indicate better performance for Micro-F1, Macro-F1, macro balanced accuracy, and sample-level Jaccard, whereas lower values indicate better performance for Hamming loss.

Bold values indicate the best-performing results.

For reference, an all-negative predictor on the 2,694-image, 22-label task has Micro-F1 0.00%, Macro-F1 0.00%, sample-level Jaccard 0.00%, Hamming loss 27.81%, and exact-match 0.00%. A seeded prevalence-matched Bernoulli reference (1,000 replicates, seed 20260824) had mean Micro-F1 40.86% (central 95% simulation interval 40.30%−41.41%), Macro-F1 27.82% (27.22%−28.46%), sample-level Jaccard 26.61% (26.16%−27.07%), Hamming loss 32.89% (32.57%−33.24%), and exact-match 0.02% (0.00%−0.07%). These are reference predictors, not model results, and are reported to contextualize the MiniMax-M2.7 floor.

MiniMax-M2.7 was retained in the primary summaries, and an eight-model sensitivity analysis assessed its influence. After excluding the MiniMax-M2.7 outlier, the prompt ordering remained unchanged. Mean Micro-F1 values were 53.52% for DP, 53.63% for KGP, and 54.53% for KGRP. Mean Macro-F1 values were 36.46%, 37.37%, and 37.77%, and mean sample-level Jaccard values were 38.76%, 38.77%, and 39.72%. Macro balanced accuracy also followed the same order (59.84%, 60.33%, and 60.55%), Hamming loss decreased from 26.21% under DP to 25.25% under KGRP, and parse success remained near ceiling after outlier removal (99.81%, 99.78%, and 99.71%). The modest mean change persisted after exclusion, but this sensitivity analysis does not remove the model-specific heterogeneity or the limitations of image-level inference.

The conflict-resolution sensitivity analysis retained the direction of the mean KGRP-vs.-DP changes (Supplementary Table S13). On all 2,694 images, the Micro-F1 contrast was +0.939 percentage points under the archived benchmark-frequency rule, +0.930 with fixed ontology-order priorities, and +0.932 without conflict removal. The corresponding Macro-F1 contrasts were +1.190, +1.100, and +1.096 points; sample-level Jaccard contrasts were +0.866, +0.854, and +0.858 points; and Hamming-loss contrasts were −0.850, −0.845, and −0.851 points. The sign of each model's KGRP-minus-DP Micro-F1 change was unchanged across these policies.

Frequencies estimated from the 2,154 training images produced the same pairwise priorities for all 12 rules and exactly the same voted predictions as the benchmark-frequency policy. On the held-out 270-image subset, mean KGRP-minus-DP Micro-F1 changes were +0.834 points with either frequency rule, +0.832 with fixed ontology-order priorities, and +0.834 without conflict removal. The fixed-order and no-removal policies changed voted outputs in 206 and 185 of the 72,738 full-set image-model-prompt combinations, respectively. These results support stability of the modest aggregate contrast to the tested policies, without establishing an optimal clinical conflict-resolution rule or patient-independent performance.

Under KGRP, several labels had substantial reference support but low recall in the canonical majority-voted predictions. Thin tongue had support of 506 images, mean F1 of 5.28% (95% image-bootstrap percentile interval, 4.48%−6.10%), precision of 37.47%, and recall of 3.12%. Blue-purple tongue had support of 1,102 images, mean F1 of 12.65% (11.36%−13.99%), precision of 70.01%, and recall of 7.53%. Gray-black tongue coating had support of 576 images, mean F1 of 10.31% (8.05%−12.43%), precision of 80.52%, and recall of 5.77%. These summaries indicate low detection sensitivity and do not identify its cause.

Among selected low-prevalence labels, no tongue coating (3.75%) had mean precision of 27.90% (95% image-bootstrap percentile interval, 19.14%−35.96%) and recall of 5.50% (3.53%−7.72%). Corresponding estimates were 44.64% (37.34%−51.76%) and 7.86% (6.45%−9.27%) for pale tongue (10.02%), and 47.57% (39.40%−55.17%) and 25.20% (22.58%−27.86%) for peeled coating (9.99%). Supplementary Table S7 reports these exploratory estimates; their intervals do not account for unmeasured within-participant image dependence.

### 4.3 Repeated-generation stability and drift

Across all 72,738 image-model-prompt combinations, the archive contained 217,437 generation records and 777 missing slots. There were 3 combinations with *Gi* = 0, 383 with *Gi* = 1, 2 with *Gi* = 2, and 72,350 with *Gi* = 3 (Supplementary Table S14). The three zero-generation records were for one Qwen3.6-Plus image under the three prompts; each supplied one cached empty set to canonical scoring. The scorer therefore used 217,440 vectors: 386 combinations had *Ki* = 1, 2 had *Ki* = 2, and 72,350 had *Ki* = 3. All 27 archived stability denominators matched these reconstructed counts. Cached empty sets are not counted as successful generations in the raw audit.

Table 4 summarizes repeated-generation behavior on all 2,694 images. For the canonical post-processed outputs, mean pairwise Jaccard consistency decreased from 70.97% under DP to 70.03% under KGRP, and Mean E_consistent decreased from 26.02% to 23.66%. Mean E_dynamic increased from 2.73% to 3.07%. Post-processed E_single was zero under all three prompts because the evaluator applied deterministic mutual-exclusion rules. These observations describe the evaluated pipeline, including parsing, rule handling, and the scorer's representation of failed outputs, rather than intrinsic model stability. Three generations provide only a preliminary description of repeated-output variation.

**Table 4:** Repeated-generation stability and raw-output conflict audit by prompt strategy.

Metric	DP (%)	KGP (%)	KGRP (%)
Mean pairwise Jaccard consistency	70.97	70.29	70.03
Mean E_consistent	26.02	24.84	23.66
Mean E_dynamic	2.73	2.75	3.07
Mean raw conflict incidence	0.382	0.747	0.070
Mean post-processed E_single	0.00	0.00	0.00
Mean raw E_single	0.035	0.070	0.007
Mean post-audit conflict incidence	0.000	0.000	0.000

Values are equal-weight means across nine models. The original stability metrics use canonical post-processed attempt vectors. Added raw-audit rates use successfully parsed generations and the same 12 mutual-exclusion rules; parse failures are excluded from their denominators (Supplementary Table S12).

Figure 7 summarizes the range of attempt-level performance metrics within each model-prompt condition, complementing the label-set consistency measures in Table 4.

**Figure 7:**
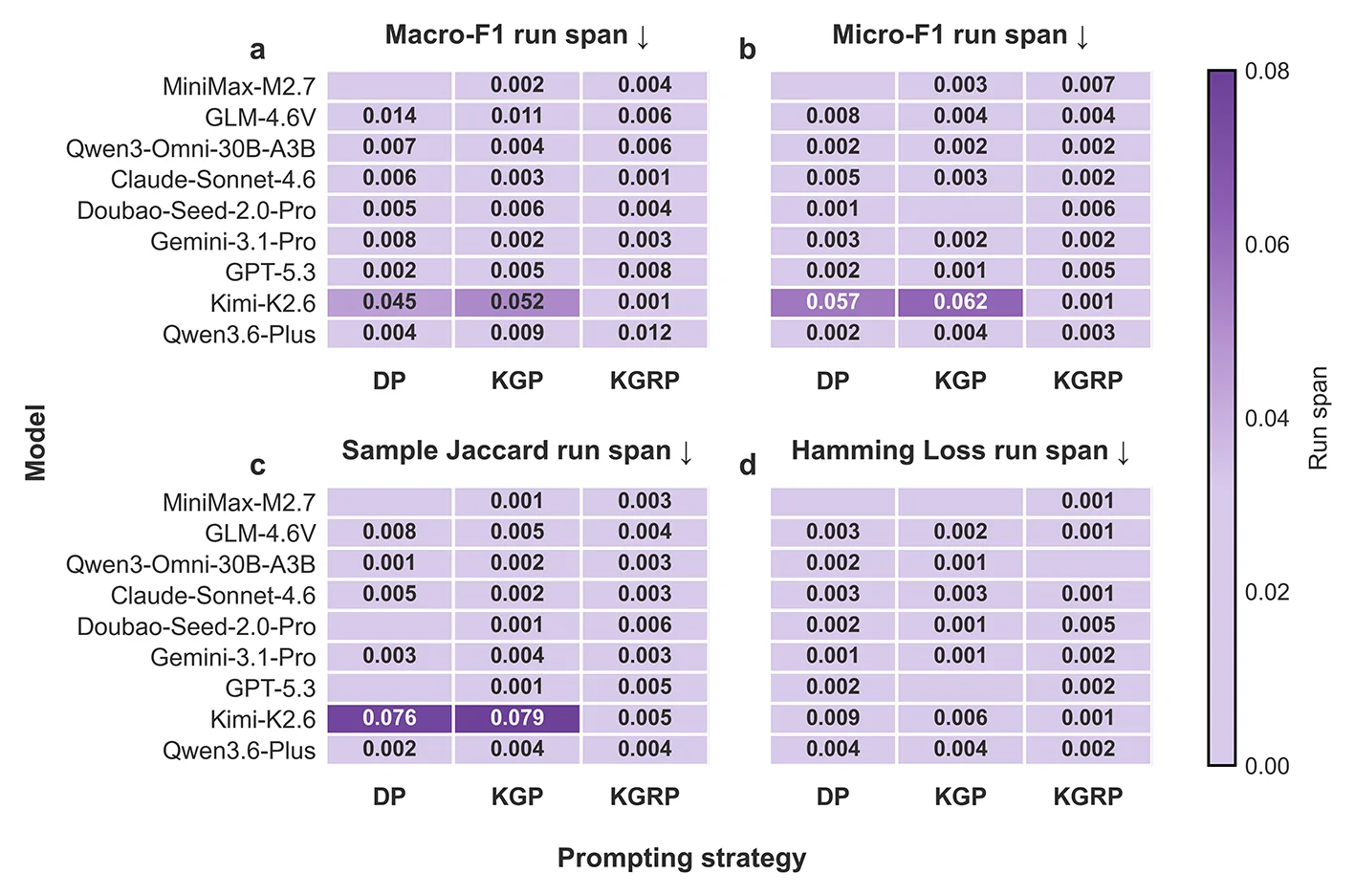
Attempt-level metric ranges under each model-prompt condition. **(a–d)** show the maximum minus minimum values of Macro-F1, Micro-F1, sample-level Jaccard, and Hamming loss across the available attempt indices. Smaller ranges indicate less variation in aggregate scores. Results use canonical post-processed predictions from the 2,694-image benchmark; missing generation records may cause the evaluated image counts to differ across attempt indices.

The raw-output audit identified 851 generations with at least one mutual-exclusion violation among 194,813 successfully parsed generations (0.437%), corresponding to 953 violated rule checks. After pooling parsed generations across models, conflict incidence was 277/65,180 (0.425%) for DP, 524/64,652 (0.810%) for KGP, and 50/64,981 (0.077%) for KGRP. Table 4 instead reports equal-weight means of the model-specific rates. The lower observed raw conflict incidence under KGRP is descriptive; the post-processed zero values demonstrate deterministic rule enforcement rather than independent model mastery of the ontology. Supplementary Table S12 provides all 27 model-prompt conditions, including 22,624 parse failures, 3,181 valid empty normalized outputs, and 777 generation slots without a retained generation record. These categories are not treated as successful non-empty, conflict-free responses.

### 4.4 Comparison with supervised baselines

On the same 270 test images, the three supervised baselines had higher F1 and Jaccard scores and lower Hamming loss than all nine KGRP LMMs. KGRP Micro-F1 ranged from 0.48% to 59.11%, compared with 68.63% to 73.00% for the supervised baselines in Table 1. These comparisons describe the image-level split; participant overlap between training and test subsets could not be excluded.

Figure 3 shows that exploratory label-level comparisons identified 31 positive KGRP-minus-baseline F1 differences among 594 comparisons, corresponding to 27 unique model–label pairs. These descriptive differences do not establish label-specific superiority, clinical benefit, or generalization to independent patients.

### 4.5 Exact-match failure burden

Using exact agreement between the majority-voted prediction and the full reference label set as the criterion, no model-prompt combination achieved an exact match for 2,552 of the 2,694 images. When results were grouped by prompt strategy, none of the nine LMMs achieved an exact match for 2,632 images under DP, 2,618 images under KGP, and 2,607 images under KGRP, representing 97.70%, 97.18%, and 96.77% of the benchmark images, respectively. KGRP therefore modestly reduced the number of images for which no evaluated model achieved an exact match.

These counts describe exact-match coverage across the evaluated model-prompt combinations, not patient-level clinical failure rates. Figure 8 illustrates selected images for which no combination recovered the complete reference label set. The examples are qualitative and do not establish the prevalence or causal contribution of individual error sources.

**Figure 8:**
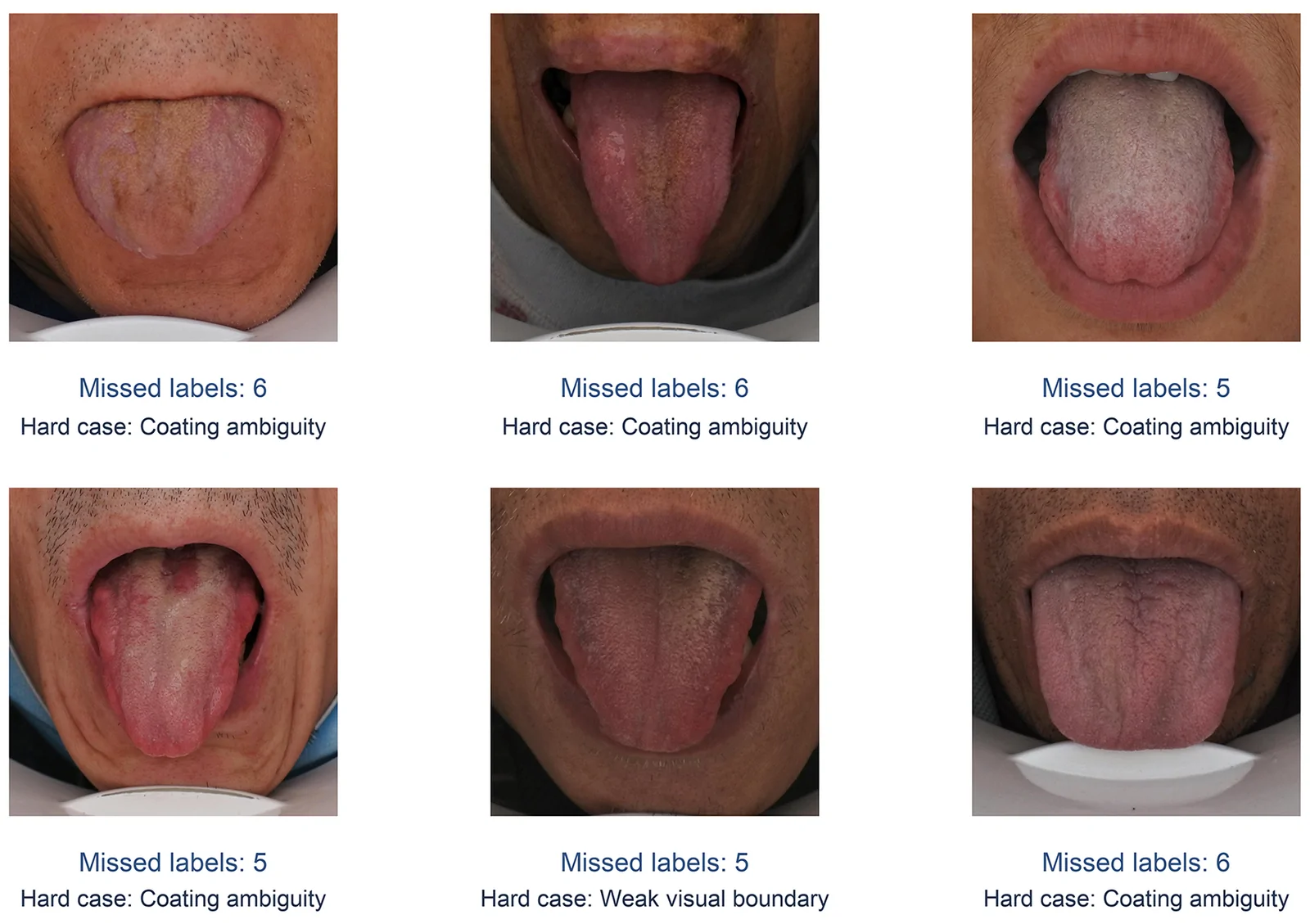
Representative images drawn from the persistent failure subset. The accompanying annotations summarize the number of missed labels and visual features noted during qualitative review.

## 5 Discussion

This study shows that prompt construction can alter the observed behavior of the evaluated API-accessed LMMs in TCM tongue diagnosis. KGRP produced modest increases in the fixed nine-model mean scores, while model-specific estimates and median/IQR summaries showed heterogeneous responses. The exploratory image-bootstrap intervals are conditional on the archived benchmark configuration and an unverified image-independence assumption. They do not establish that KGRP is generally superior across a population of LMMs. DP, KGP, and KGRP therefore remain useful controlled evaluation conditions, but their effects should be interpreted within the specific models, outputs, and scoring protocol examined here.

Prompt sensitivity has also been examined in clinical natural language processing and multimodal evaluation (Sivarajkumar et al., 2024; Ismithdeen et al., 2025). TongueBench extends this line of inquiry to multi-label tongue-image assessment under a fixed ontology. In this setting, prompt effects must be evaluated alongside parsing behavior, rule enforcement, and the recovery of coexisting labels.

The supervised comparison used the same 270 test images for both model families, resolving the original mismatch in image scope. Nevertheless, the supervised split was performed at the image level, and participant linkage was unavailable. Similar images acquired from the same participant may occur in both training and test subsets, potentially inflating supervised scores. Thus, the observed aggregate ordering describes this split and should not be extrapolated to patient-independent relative performance. The exploratory label-level differences in Figure 3 also require this qualification and provide no basis for claims of clinical superiority.

The stability results depend on the evaluation stage. Raw parsed outputs contained ontology violations, which deterministic post-processing removed. A post-processed E_single_ of zero therefore indicates enforcement of the specified rules. KGRP had fewer observed raw conflicts than DP or KGP, but slightly lower mean pairwise and exact repeated-output consistency than DP. These measures capture different aspects of output behavior and use different denominators. Parsing failures, valid empty sets, missing records, and normalization affect their interpretation; they are not direct measures of faithful reasoning or intrinsic API stochasticity.

The additional sensitivity analysis addresses a separate evaluator dependence: the use of benchmark label frequencies to resolve conflicts. The mean prompt contrast and its model-specific Micro-F1 directions persisted with a frequency-independent fixed order and without conflict removal. Training-only frequencies also yielded identical decisions on the held-out test subset because all pairwise priorities were unchanged. The original scores nevertheless remain outputs of a benchmark-informed pipeline. These checks show that the observed mean contrast was not driven by the tested frequency priorities; they do not validate the original use of test-set label information, exclude sensitivity to other rules, or establish a clinically appropriate priority ordering.

The ontology-constrained design makes output errors traceable to labels and explicit rules. Its auditability depends on retaining both raw and processed outputs, documenting parser behavior, and reporting the denominator used for each metric. Reporting only cleaned outputs would conceal model-generated contradictions, while reporting only average accuracy would obscure cross-model variation and parsing failures. The present results support transparent behavioral measurement without demonstrating internal visual grounding or a causal reasoning mechanism.

Model heterogeneity further argues against a universally optimal prompt strategy. KGRP improved Micro-F1 in seven LMMs but not in GPT-5.3 or Qwen3-Omni-30B-A3B. Differences in instruction-following behavior, visual calibration, safety alignment, response formatting, and decoding policies may all affect how a model responds to added domain knowledge and reflective constraints. Future benchmark reports should therefore avoid presenting one prompt as universally optimal. A benchmark-compliant approach is to report prompt construction strategy, output schema, label-normalization rules, repeated-generation behavior, model-level response heterogeneity, and label-level effects together.

The exact-match analysis identifies a further limitation of the evaluated systems: recovering the complete label set remained difficult even when aggregate F1 improved. Even after KGRP, complete label-set reconstruction was rare. This is not surprising because most images contained 5–7 positive labels, and the strict exact-match metric penalizes every missing or extra label. This finding concerns complete reconstruction of the annotated tongue signs. The benchmark did not evaluate how individual label errors would affect a clinician's diagnosis or treatment decisions.

TongueBench treats the prompt, diagnostic ontology, output schema, normalization rules, and repeated-generation evaluation as components of a measurable protocol. Its contribution is a transparent framework for inspecting observed performance and failure modes across the evaluated LMMs. Reproducible local scoring is supported by retained predictions and evaluation artifacts, whereas exact replication of historical API inference is limited by incomplete parameter records and endpoint changes. A non-causal summary of observed failure patterns is provided in Supplementary Table S5.

Proprietary API models do not disclose complete pretraining corpora or update histories. The supplied archive does not establish whether TongueBench images, source data, derivative images, or related tongue-image corpora were available before each model run. Pretraining exposure and later model updates therefore cannot be ruled out, which limits the interpretation of the term zero-shot.

## 6 Limitations

This study has several limitations. First, the acquisition protocol included repeated images per participant, but validated participant-to-image links and patient counts are unavailable. The image-level train/validation/test split cannot exclude participant overlap, and within-participant image correlation may produce optimistic supervised test scores. Second, image-level bootstrap intervals may be too narrow under this dependence. They describe a fixed set of nine models and archived outputs, not a population of LMMs or independent patients. Third, the original independent expert annotations and adjudication logs were unavailable. Inter-rater agreement could therefore not be estimated, leaving the reliability of the consensus reference standard quantitatively uncharacterized. Fourth, three generations provide only a preliminary stability probe, and effective historical decoding parameters and provider defaults could not be verified. Missing generation records, parse failures, and valid empty outputs further constrain interpretation of output reliability. Fifth, zero residual ontology violations after processing reflect deterministic evaluator rules; they do not independently establish model ontology consistency. The archived conflict-resolution policy used frequencies from the benchmark reference workbook. Although fixed-order, training-frequency, and no-removal analyses preserved the main descriptive contrast, this does not make the original pipeline independent of benchmark-derived priors or validate a clinical priority rule. The training-frequency analysis is held out only on the 270-image test subset. Finally, incomplete acquisition, color-calibration, and demographic records prevent assessment of device robustness, demographic fairness, and cross-institutional generalizability. The benchmark does not establish visual grounding or clinical utility.

## 7 Conclusion

TongueBench provides a prompt-controlled protocol for evaluating observable multi-label reconstruction and output behavior in API-accessed LMMs. KGRP produced a modest improvement in the mean scores of the nine evaluated models, with heterogeneous model-specific changes and persistent complete-label reconstruction failures. The modest mean contrast persisted under the tested alternatives to benchmark-frequency conflict resolution. Raw-output conflicts, post-processing effects, and denominators for retained and missing generations must be reported separately. The same-image supervised comparison and exploratory bootstrap summaries remain limited by unverified patient independence. These findings support transparent benchmarking of the evaluated systems, but do not establish universal prompt superiority, patient-level generalizability, or readiness for autonomous diagnosis.

## Data Availability

The datasets presented in this study can be found in online repositories. The names of the repository/repositories and accession number(s) can be found at: Zenodo: doi: 10.5281/zenodo.20701289. The source code is publicly available at https://github.com/mengyiha/TongueBench-Open.
