# Supplementary material for: TongueBench: performance of prompt-engineered large multimodal models in Traditional Chinese Medicine tongue diagnosis
Source: Front Artif Intell. 2026 Oct 21;9:1943004. doi: 10.3389/frai.2026.1943004 (PMC13731349; doi:10.3389/frai.2026.1943004)
Supplement: Supplementary file 1 [file Data_Sheet_1.pdf]

TongueBench Supplementary Material

TongueBench: Performance of Prompt-Engineered Large Multimodal Models in Traditional Chinese Medicine Tongue Diagnosis

This supplement provides the archived prompts, scoring details, and supplementary tables for the revised TongueBench manuscript. The full prompt benchmark uses 2,694 images; the primary comparison with supervised baselines uses the same 270 test images. Participant-level independence could not be established.

Supplementary prompts

The archived prompt and protocol text below is reproduced without changing its substantive wording. Protocol requirements describe the intended design; they do not establish that historical provider settings, retry decisions, or hard-case trigger sources were retained or verified. The observed scoring procedures and unavailable metadata are distinguished in the Supplementary Methods and Table S1.

Archived artifact: Tongue_Prompt_20260108/SYSTEM_PROMPT.md

SYSTEM_PROMPT
你是一个舌诊图像识别系统，能够通过图像对中医舌诊做出精确识别。
任务：基于舌象图像中的可见视觉特征，对预定义 22 个标签进行判断。
全局约束：
1. 仅依据图像可见特征判断，只按格式输出结果，不得进行任何解释和说明。
2. 只允许舌部区域作为判断目标；不得依据嘴唇、牙齿、口腔、文字水印、边框、背景等作判断。
3. 输出必须且只能是一个合法 JSON 对象，不得输出额外文本、注释或 Markdown。
4. 顶层键顺序固定为：`labels`、`hard_case`、`confidence`。
5. `labels` 的值只允许 `0` 或 `1`；`hard_case` 只允许 `true` 或 `false`；`confidence` 只允许 `"high"`、`"medium"`、`"low"`。
6. 必须输出全部 22 个标签键，每个键仅出现一次。
7. `hard_case` 定义为“高难样本”，包含两类：图像质量困难 或 标签判别争议；不等价于“仅低质量图像”。
8. 证据不足、图像质量不佳或标签不稳定时，对应标签必须输出 `0`，不得猜测。
9. `confidence` 规则为：`"high"`：无明显质量问题，核心维度均可稳定判断。 `"medium"`：存在轻度质量问题或局部不稳定，但整体可判。`"low"`：存在明显质量问题或多个维度不稳定。
10. `hard_case` 规则：若舌体主体可见面积明显不足（约低于 60%）或存在严重失焦/严重曝光异常/严重色偏导致核心维度无法判断，则输出进入保守模式：`labels` 全部置 `0`、`hard_case=true`、`confidence="low"`。
11. 示例仅用于格式演示，不可复用示例中的标签取值。

Archived artifact: Tongue_Prompt_20260108/User_Prompt/Benchmark_Protocol.md

Prompt Benchmark Protocol (3 Groups)
本项目仅保留三组提示词，用于可复现实验：
1. A0_Control_Direct
2. A1_Knowledge_Direct
3. A2_Knowledge_Reflection_Strict
消融变量定义
- A0 -> A1：仅增加"标签定义知识"。
- A1 -> A2：仅增加"反思流程模块"（反证审查 + 互斥冲突审查 + 保守回退 + hard_case 判定）。
本体声明（论文必须写明）
1. `舌色红绛` 为研究自定义合并标签，对应传统"红舌 + 绛舌"合并。
2. `舌色淡白`、`舌色淡红`、`舌色红绛` 为舌色主轴标签，三者互斥；`舌色青紫` 可与三者之一共存，也可单独出现。
3. 论文方法部分必须给出上述声明及与传统术语的映射说明。
固定项（所有组必须一致）
1. 标签集合固定为 22 标签，顺序固定。
2. 输出 JSON schema 固定为：`labels`、`hard_case`、`confidence`。
3. 推理超参数固定（temperature、top_p、max_tokens、seed）。
4. 数据划分固定（train/val/test 或固定评测集）。
5. 评测脚本与后处理固定。
6. 同一批样本、同一调用重试策略、同一 JSON 解析器。
7. `hard_case` 规则固定为"质量困难或标签争议"的并集定义。
hard_case 操作性定义
1. hard_case 不是"仅低质量图片"，而是"高难样本"。
2. hard_case 触发来源分两类：
  - 质量困难（Q-hard）：可见面积不足、失焦、遮挡、曝光异常、严重色偏。
  - 判别争议（D-hard）：反证或冲突回退后仍无法稳定确定关键标签。
3. 输出端只保留布尔值 `hard_case`；评测日志需额外记录触发来源（Q-hard / D-hard / both）。
强制报告指标（不再是"建议"）
1. JSON 解析成功率（Parse Success Rate）与无效率（Invalid Output Rate）。
2. Micro-F1。
3. Macro-F1。
4. 每标签 Precision / Recall / F1（含支持度）。
5. Hamming Loss。
6. 严格子集准确率（Subset Accuracy）。
7. Hard-case 子集上的 Micro-F1 / Macro-F1（用于评估鲁棒性）。
统计显著性与稳定性
1. 每组至少 3 次独立运行，报告均值和标准差。
2. 对 A1 vs A0、A2 vs A1 做配对显著性检验（推荐 bootstrap 95% CI）。
3. 若提升不显著（置信区间跨 0），不得宣称"有效提升"。
结果记录要求
1. 错例分析按"假阳性来源/假阴性来源/冲突回退样例"分类。
2. A2 额外报告：
  - hard_case 比例
  - hard_case 与非 hard_case 的性能差异
  - hard_case 来源比例（Q-hard / D-hard / both）
  - 冲突回退触发比例
3. 全部实验需记录 prompt 版本号与时间戳，保证可追溯。

Archived artifact: Tongue_Prompt_20260108/User_Prompt/A0_Control_Direct.md

A0_Control_Direct
标签集合（固定顺序）：
["舌嫩","舌老","舌胖","舌瘦","舌上点刺","舌面裂纹","舌边齿痕","舌上瘀斑","舌色淡白","舌色淡红","舌色红绛","舌色青紫","苔无","苔薄","苔厚","苔滑润","苔燥","苔腐腻","苔剥落","苔色白","苔色黄","苔色褐黑"]
输出 JSON 示例：

```
{
  "labels": {
    "舌嫩": 0,
    "舌老": 0,
    "舌胖": 1,
    "舌瘦": 0,
    "舌上点刺": 0,
    "舌面裂纹": 0,
    "舌边齿痕": 1,
    "舌上瘀斑": 0,
    "舌色淡白": 0,
    "舌色淡红": 1,
    "舌色红绛": 0,
    "舌色青紫": 0,
    "苔无": 0,
    "苔薄": 1,
    "苔厚": 0,
    "苔滑润": 0,
    "苔燥": 0,
    "苔腐腻": 0,
    "苔剥落": 0,
    "苔色白": 1,
    "苔色黄": 0,
    "苔色褐黑": 0
  },
  "hard_case": false,
  "confidence": "medium"
}
```

Archived artifact: Tongue_Prompt_20260108/User_Prompt/A1_Knowledge_Direct.md

A1_Knowledge_Direct
目标：增加标签定义知识。
标签定义
舌体形态
- 舌嫩：舌体较柔嫩，纹理细腻，表面相对光滑。
- 舌老：舌体纹理较粗，质地偏老，皱缩或粗糙感较明显。
- 舌胖：舌体宽大肥厚，边缘膨隆。
- 舌瘦：舌体瘦薄狭长，整体偏小。
- 舌上点刺：舌面可见点状或刺点样突起。
- 舌面裂纹：舌面可见裂沟或裂纹。
- 舌边齿痕：舌边可见牙齿压痕，呈波浪或锯齿样凹陷。
- 舌上瘀斑：局部紫暗色斑点或斑片，与周围区域有明显对比。
舌体颜色
- 舌色淡白：整体偏白偏淡，红色成分不足。
- 舌色淡红：淡红或粉红，色泽相对均匀。
- 舌色红绛：整体呈较明显红色至深红色。
- 舌色青紫：整体或明显区域呈青紫、紫暗色调。
舌苔质地
- 苔无：舌面基本无可辨苔层，以舌质外露为主。
- 苔薄：苔层薄，舌质可透见。
- 苔厚：苔层较厚，较大范围遮蔽舌质。
- 苔滑润：苔面津润，光泽或湿润感明显。
- 苔燥：苔面干燥少津，欠光泽，可伴粗糙或干裂感。
- 苔腐腻：苔质黏腻或腐松，呈腻滞或豆渣样外观。
- 苔剥落：苔层局部缺失，露出下方舌质。
舌苔颜色
- 苔色白：苔色白或乳白。
- 苔色黄：苔色黄或黄褐。
- 苔色褐黑：苔色褐至灰黑。

输出 JSON 示例：


```
{
  "labels": {
    "舌嫩": 0,
    "舌老": 0,
    "舌胖": 1,
    "舌瘦": 0,
    "舌上点刺": 0,
    "舌面裂纹": 0,
    "舌边齿痕": 1,
    "舌上瘀斑": 0,
    "舌色淡白": 0,
    "舌色淡红": 1,
    "舌色红绛": 0,
    "舌色青紫": 0,
    "苔无": 0,
    "苔薄": 1,
    "苔厚": 0,
    "苔滑润": 0,
    "苔燥": 0,
    "苔腐腻": 0,
    "苔剥落": 0,
    "苔色白": 1,
    "苔色黄": 0,
    "苔色褐黑": 0
  },
  "hard_case": false,
  "confidence": "medium"
}
```

Archived artifact: Tongue_Prompt_20260108/User_Prompt/A2_Knowledge_Reflection.md

A2_Knowledge_Reflection_Strict
目标：引入严格反思流程（初判 -> 反证审查 -> 冲突审查 -> 保守回退 -> 难例判定）。
标签定义
舌体形态
- 舌嫩：舌体较柔嫩，纹理细腻，表面相对光滑。
- 舌老：舌体纹理较粗，质地偏老，皱缩或粗糙感较明显。
- 舌胖：舌体宽大肥厚，边缘膨隆。
- 舌瘦：舌体瘦薄狭长，整体偏小。
- 舌上点刺：舌面可见点状或刺点样突起。
- 舌面裂纹：舌面可见裂沟或裂纹。
- 舌边齿痕：舌边可见牙齿压痕，呈波浪或锯齿样凹陷。
- 舌上瘀斑：局部紫暗色斑点或斑片，与周围区域有明显对比。
舌体颜色
- 舌色淡白：整体偏白偏淡，红色成分不足。
- 舌色淡红：淡红或粉红，色泽相对均匀。
- 舌色红绛：整体呈较明显红色至深红色。
- 舌色青紫：整体或明显区域呈青紫、紫暗色调。
舌苔质地
- 苔无：舌面基本无可辨苔层，以舌质外露为主。
- 苔薄：苔层薄，舌质可透见。
- 苔厚：苔层较厚，较大范围遮蔽舌质。
- 苔滑润：苔面津润，光泽或湿润感明显。
- 苔燥：苔面干燥少津，欠光泽，可伴粗糙或干裂感。
- 苔腐腻：苔质黏腻或腐松，呈腻滞或豆渣样外观。
- 苔剥落：苔层局部缺失，露出下方舌质。
舌苔颜色
- 苔色白：苔色白或乳白。
- 苔色黄：苔色黄或黄褐。
- 苔色褐黑：苔色褐至灰黑。
反思流程：
1. 冲突审查：执行互斥一致性检查。
   - `舌嫩` 与 `舌老` 不可同时为 `1`。
   - `舌胖` 与 `舌瘦` 不可同时为 `1`。
   - `苔薄` 与 `苔厚` 不可同时为 `1`。
   - `苔滑润` 与 `苔燥` 不可同时为 `1`。
   - `苔无` 不可与 `苔薄`、`苔厚`、`苔滑润`、`苔燥`、`苔腐腻`、`苔剥落`、`苔色白`、`苔色黄`、`苔色褐黑` 同时为 `1`。
   - `舌色淡白`、`舌色淡红`、`舌色红绛` 不可同时为 1。
   - `舌色青紫` 可与 `舌色淡白`、`舌色淡红`、`舌色红绛`三者之一共存，也可单独出现。
2. 保守回退：
   - 冲突时仅保留证据最明确者；无法稳定排序时，冲突标签全部置 `0`。
   - 仅由单一弱证据支撑的阳性标签置 `0`。

输出 JSON 示例：

```json
{
  "labels": {
    "舌嫩": 0,
    "舌老": 0,
    "舌胖": 1,
    "舌瘦": 0,
    "舌上点刺": 0,
    "舌面裂纹": 0,
    "舌边齿痕": 1,
    "舌上瘀斑": 0,
    "舌色淡白": 0,
    "舌色淡红": 1,
    "舌色红绛": 0,
    "舌色青紫": 0,
    "苔无": 0,
    "苔薄": 1,
    "苔厚": 0,
    "苔滑润": 0,
    "苔燥": 0,
    "苔腐腻": 0,
    "苔剥落": 0,
    "苔色白": 1,
    "苔色黄": 0,
    "苔色褐黑": 0
  },
  "hard_case": false,
  "confidence": "medium"
}
```

Supplementary methods

Archived protocol and historical API settings

The model-facing prompts define conservative rollback as retaining the better-supported side of a conflict and setting both sides to zero when evidence cannot be ranked. The hard_case flag combines image-quality difficulty and label-disagreement triggers. These are requested behaviors, not independently verified records of internal model reasoning. Trigger-source fields, retry decisions, and effective historical temperature, top-p/top-k, maximum output tokens, and seed values were not consistently retained. Table S1 reports the available endpoint identifiers and timestamps; exact historical API inference cannot be reproduced. Local rescoring uses the retained predictions. No additional API generations or decoding-parameter sweeps were performed for this revision.

Canonical scoring and the raw-output audit

Canonical headline performance, label-level estimates, and repeated-generation metrics use the archived evaluate_V2.0 predictions. The raw-output audit in Tables S6 and S12 preserves the original parse-success flags. Its eligible outputs have a true archived parse flag and a parsed labels field represented as a dictionary or list. Labels are normalized, deduplicated, and restricted to the 22-label ontology before applying mutual-exclusion rules. Valid empty normalized outputs are included among parsed generations, but are reported separately from successful nonempty outputs. Failed parses and planned slots without a retained generation record are separate categories. The raw audit and canonical scoring can therefore have different parse classifications and record counts.

Mutual-exclusion rules and reference-label information

The evaluator applies 12 pairwise rules: tender versus tough tongue, enlarged versus thin tongue, thin versus thick coating, and no tongue coating versus each of thin, thick, moist, dry, rotten and greasy, peeled, white, yellow, and gray-black coating. Conflicts retain the label with greater support in the supplied reference-label workbook, with a deterministic tie-break. The frequency priorities were not restricted to the supervised training subset. Post-processed results therefore include benchmark-derived label-frequency information. Broader checks in the KGRP prompt, including the primary tongue-color axis and moist-versus-dry coating, are model-facing instructions rather than additional evaluator rules. Sensitivity analyses additionally use fixed ontology-order priorities, training-only frequencies, and no conflict removal (Table S13). The fixed order is a technical comparator and does not assert clinical priority.

Retained attempts and denominators

Three generations were requested per image and model-prompt condition. G_i denotes the retained generation-record count and K_i the scoring-vector count. Each retained generation supplies a vector, including zeros for unusable outputs. For three Qwen3.6-Plus records with no generations, the archived scorer read an empty cached voted-label set: these have $G_i = 0$ and $K_i = 1$. Otherwise $K_i = G_i$. Voting requires strictly more than $K_i/2$ positive votes; thresholds for $K_i = 1$, 2, and 3 are 1, 2, and 2. Missing slots are not padded to three attempts, and cached vectors are not generations or parsed responses. A $K_i = 0$ case would yield an all-zero vote but was not observed after cache reading. Pairwise Jaccard averages over $K_i \geq 2$ images with empty-empty similarity 1. $E_{consistent}$ uses all available pairs, with denominator $\Sigma_i K_i(K_i-1)/2$. Canonical E_{single} uses $12\Sigma_i K_i$ checks; $E_{dynamic}$ uses 12 times the number of $K_i \geq 1$ images, with no drift for $K_i = 1$. Raw-audit E_{single} uses only eligible parsed generations. All 27 canonical stability denominators match this reconstruction. Table S14 distinguishes records and scored vectors; S12 retains the raw parsing categories.

Paired uncertainty and descriptive summaries

Bootstrap estimates use 1,000 image-level resamples with seed 20260823. The same image indices are used across all nine models and prompt strategies. Metrics are recomputed within each model before averaging model-specific contrasts with equal weights. Percentile limits are the 2.5th and 97.5th percentiles. Table S7 applies the same shared-image resampling scheme to model-averaged label precision, recall, and F1, with zero-denominator values set to zero. Tables S10 and S11 describe the fixed nine evaluated models, not a broader model population. Table S10 quartiles use linear interpolation; paired-change medians are computed from the nine within-model differences. Missing participant identifiers prevent a participant-cluster bootstrap, so all image-bootstrap intervals are conditional and exploratory and may be too narrow. No multiplicity-corrected superiority claim is made.

Image scope and expert reference labels

The supervised split contains 2,154 training, 270 validation, and 270 test images, selected at image level. Table S2 matches LMM predictions to the same 270 test-image identifiers. Repeated acquisition per participant and missing validated participant-to-image linkage prevent verification of patient separation. Potential participant overlap may inflate supervised test performance. Only final consensus/adjudicated expert labels were retained; independent annotations and detailed adjudication records are unavailable, so inter-rater agreement cannot be reconstructed and no human-performance ceiling is inferred.

Supplementary Table S1. Archived API model and run metadata.

Model identifier	Endpoint host	Recorded access date(s)
MiniMax-M2.7	bm.uf.yigechengzi.com	20260410_101656; 20260418_210042; 20260419_211941; 20260420_174237
Qwen/Qwen3-Omni-30B-A3B-Thinking	api.siliconflow.cn	20260419_220457
anthropic/claude-sonnet-4.6	openrouter.ai	20260422_191751; 20260423_173401
doubao-seed-2-0-pro-260215	ark.cn-beijing.volces.com	20260420_173721; 20260421_113302
gemini-3.1-pro-preview-nothinking	ai.biutag.com	20260430_222027
moonshotai/kimi-k2.6	openrouter.ai	20260421_152341
openai/gpt-5.3-chat	openrouter.ai	20260428_120724
qwen/qwen3.6-plus:free	openrouter.ai	20260406_183204
zai-org/GLM-4.6V	api.siliconflow.cn	20260418_124822

Each model was evaluated on 2,694 images under DP, KGP, and KGRP, with three requested generations per image and prompt. Identifiers and timestamps are retained archive strings. Effective decoding settings and provider update histories could not be verified. Raw recorded and missing counts are provided in Table S12A.

Supplementary Table S2. KGRP LMM performance on the same 270 test images used by the supervised baselines.

Model	Micro-F1	Macro-F1	Sample F1	Sample Jaccard	Hamming loss	Exact match
Doubao-Seed-2.0-Pro	59.11%	43.85%	59.04%	44.10%	24.55%	0.74%
Gemini-3.1-Pro	57.97%	44.82%	57.93%	43.12%	25.00%	0.74%
Claude-Sonnet-4.6	55.73%	41.47%	55.50%	40.58%	28.03%	0.37%
Kimi-K2.6	55.20%	39.48%	55.23%	40.49%	25.25%	1.11%
Qwen3.6-Plus	54.85%	37.72%	54.72%	39.90%	26.55%	1.48%
GPT-5.3	53.03%	33.79%	53.15%	38.46%	24.24%	0.74%
GLM-4.6V	50.23%	27.58%	50.65%	36.49%	24.12%	0.37%
Qwen3-Omni-30B-A3B	48.69%	28.37%	48.89%	34.76%	26.46%	0.74%
MiniMax-M2.7	0.48%	0.28%	0.28%	0.18%	27.86%	0.00%

All values are percentages. This is the primary LMM-versus-supervised comparison subset, consistent with manuscript Table 4 and Figure 7; the full prompt benchmark contains 2,694 images. The supervised split is image-based and patient-level separation is unverified. Figure 7 summarizes 31 positive observed F1 differences among 594 comparisons (22 labels x 9 LMMs x 3 baselines), corresponding to 27 unique LMM-label pairs. These are exploratory differences, not evidence of label-specific clinical superiority.

Supplementary Table S3. Canonical model-by-prompt results on the 2,694-image benchmark.

Model	Prompt	Micro-F1	Macro-F1	Macro balanced accuracy	Sample Jaccard	Hamming loss	Pairwise Jaccard consistency	Parse success
MiniMax-M2.7	DP	0.00%	0.00%	50.00%	0.00%	27.81%	99.55%	8.85%
MiniMax-M2.7	KGP	0.04%	0.02%	50.00%	0.02%	27.81%	98.32%	6.22%
MiniMax-M2.7	KGRP	0.36%	0.18%	49.99%	0.15%	27.80%	90.68%	10.79%
Qwen3-Omni-30B-A3B	DP	49.27%	28.97%	54.37%	35.08%	27.97%	53.56%	99.79%
Qwen3-Omni-30B-A3B	KGP	48.72%	29.86%	55.83%	34.69%	26.01%	51.01%	99.73%
Qwen3-Omni-30B-A3B	KGRP	48.08%	29.26%	55.45%	33.92%	26.79%	48.95%	99.63%
Claude-Sonnet-4.6	DP	55.38%	40.52%	61.05%	40.25%	28.13%	75.01%	99.94%
Claude-Sonnet-4.6	KGP	54.84%	40.88%	60.96%	39.59%	29.35%	75.11%	99.88%
Claude-Sonnet-4.6	KGRP	55.67%	40.79%	61.20%	40.42%	28.12%	73.55%	99.91%
Doubao-Seed-2.0-Pro	DP	57.40%	43.21%	63.67%	42.28%	27.12%	81.85%	100.00%
Doubao-Seed-2.0-Pro	KGP	58.03%	44.17%	64.57%	42.81%	25.98%	79.42%	100.00%
Doubao-Seed-2.0-Pro	KGRP	59.49%	45.41%	64.98%	44.44%	24.06%	77.65%	100.00%
Gemini-3.1-Pro	DP	55.48%	45.56%	65.68%	40.35%	26.07%	77.00%	100.00%
Gemini-3.1-Pro	KGP	56.44%	45.93%	65.54%	41.14%	25.89%	76.13%	100.00%
Gemini-3.1-Pro	KGRP	59.12%	47.16%	66.29%	44.04%	24.00%	81.45%	100.00%
Kimi-K2.6	DP	53.06%	38.07%	60.58%	37.95%	26.55%	50.92%	99.28%
Kimi-K2.6	KGP	53.52%	38.85%	60.64%	38.27%	25.33%	50.11%	99.00%
Kimi-K2.6	KGRP	55.41%	40.39%	61.19%	40.45%	24.85%	59.29%	98.90%
GPT-5.3	DP	54.40%	31.74%	57.87%	39.86%	22.13%	75.89%	100.00%
GPT-5.3	KGP	53.22%	33.95%	58.95%	38.59%	24.52%	76.96%	100.00%
GPT-5.3	KGRP	53.29%	33.16%	58.52%	38.59%	23.87%	74.68%	100.00%
Qwen3.6-Plus	DP	54.48%	39.65%	61.43%	39.52%	27.42%	62.23%	99.86%
Qwen3.6-Plus	KGP	54.56%	38.86%	60.88%	39.40%	27.09%	61.30%	99.94%
Qwen3.6-Plus	KGRP	55.18%	39.24%	60.98%	40.13%	26.42%	59.50%	99.94%
GLM-4.6V	DP	48.66%	23.94%	54.09%	34.81%	24.28%	62.67%	99.59%
GLM-4.6V	KGP	49.72%	26.43%	55.27%	35.67%	23.81%	64.25%	99.70%
GLM-4.6V	KGRP	49.98%	26.78%	55.77%	35.75%	23.92%	64.55%	99.28%

Accuracy metrics use majority-voted predictions. Pairwise Jaccard consistency uses retained attempt-level predictions, and parse success uses canonical generation records. All values are percentages. Larger values are favorable except for Hamming loss. Empty or failed outputs can increase apparent repeated-output consistency. Bold text denotes table headers only.

Supplementary Table S4. Descriptive summary of prompt-associated results.

Prompt	Added content	Observed result	Interpretation limit
DP	Task instruction, 22-label inventory, and structured output schema	Highest Micro-F1 in 2 of 9 models; highest nine-model mean pairwise and exact repeated-output consistency.	Lower mean F1 and Jaccard than KGRP; repeated-output consistency does not establish useful or correct predictions.
KGP	DP plus fixed TCM label definitions across four ontology domains	Small mean changes relative to DP; nine-model Macro-F1 increased from 32.41% to 33.22%.	Changes were model- and label-dependent; complete label-set reconstruction remained uncommon.
KGRP	KGP plus contradiction review, mutual-exclusion checks, conservative rollback, and hard-case instructions	Highest Micro-F1 in 7 of 9 models; mean Micro-F1 48.51%, compared with 47.57% under DP.	Not uniformly beneficial; conditional image-bootstrap inference, low exact-match coverage, and slightly lower mean repeated-output consistency limit interpretation.

This summary refers to the full 2,694-image benchmark and the fixed nine models. It does not identify causal mechanisms or establish a generally optimal prompt.

Supplementary Table S5. Qualitative descriptions of observable error patterns.

Error type	Observable pattern	Possible interpretation, not causally tested
Visual Feature Omission	One or more expert-adjudicated tongue signs are absent from the predicted label set, even when other signs are recovered	Subtle visual boundaries, low-salience or rare signs, and competition among 5–7 concurrent positive labels in most images
Visual Feature Hallucination	The model adds an ontology label that is not present in the expert reference set, reducing Jaccard and exact-match performance	Overinterpretation of ambiguous color or texture cues, or reflective reconsideration that expands the candidate-label set
Label Confusion	The prediction shifts between visually or semantically adjacent categories, such as body-color, coating-thickness, or moisture alternatives	Fine-grained category boundaries, imbalanced label support, and incomplete alignment between visual evidence and ontology definitions
Multi-label Incompleteness	Several labels are predicted correctly, but the complete expert label set is not reconstructed	High label cardinality and the cumulative burden of missing or adding any one sign under the strict exact-match criterion
Ontology Violation	Mutually exclusive labels are selected within one output or opposite sides of a rule are selected across repeated generations	Raw violations may reflect incomplete application of the prompt instructions. Zero residual within-round violations reflect evaluator-side enforcement; cross-round drift remains a separate measure.
Prompt Sensitivity	A label or model changes performance or output composition across DP, KGP, and KGRP	Differences in instruction following, use of injected knowledge, response revision, formatting behavior, and model-specific decoding policies

These categories describe patterns discussed in the manuscript and illustrated by selected examples. They are not a newly coded or quantified error-analysis dataset. Hypotheses do not demonstrate internal visual grounding, reasoning faithfulness, or clinical consequences.

Supplementary Table S6. Mini-Max raw parsing counts and canonical scoring results.

Model	Prompt	Raw parse failures	Raw valid empty	Raw failure rate	Canonical parse success	Voted Micro-F1
MiniMax-M2.7	DP	7369	695	91.18%	8.85%	0.00%
MiniMax-M2.7	KGP	7584	429	93.84%	6.22%	0.04%
MiniMax-M2.7	KGRP	7214	476	89.26%	10.79%	0.36%

Each prompt has 8,082 retained raw generation records. The raw failure rate divides original parse failures by those records. Raw valid empty outputs are successfully parsed generations with an empty normalized label set. Canonical parse success and voted Micro-F1 retain the headline scoring definition; raw and canonical classifications are not interchangeable.

Supplementary Table S7. Canonical label-level estimates under KGRP on 2,694 images.

Label	Metric	Prevalence	Estimate	95% lower	95% upper
Thin tongue	Precision	18.78%	0.3747	0.2898	0.4662
Thin tongue	Recall	18.78%	0.0312	0.0261	0.0369
Thin tongue	F1	18.78%	0.0528	0.0448	0.0610
Blue-purple tongue	Precision	40.91%	0.7001	0.6473	0.7450
Blue-purple tongue	Recall	40.91%	0.0753	0.0672	0.0836
Blue-purple tongue	F1	40.91%	0.1265	0.1136	0.1399
Gray-black tongue coating	Precision	21.38%	0.8052	0.7696	0.8321
Gray-black tongue coating	Recall	21.38%	0.0577	0.0445	0.0701
Gray-black tongue coating	F1	21.38%	0.1031	0.0805	0.1243
Pale tongue	Precision	10.02%	0.4464	0.3734	0.5176
Pale tongue	Recall	10.02%	0.0786	0.0645	0.0927

Pale tongue	F1	10.02%	0.1166	0.0952	0.1375
No tongue coating	Precision	3.75%	0.2790	0.1914	0.3596
No tongue coating	Recall	3.75%	0.0550	0.0353	0.0772
No tongue coating	F1	3.75%	0.0874	0.0564	0.1187
Peeled coating	Precision	9.99%	0.4757	0.3940	0.5517
Peeled coating	Recall	9.99%	0.2520	0.2258	0.2786
Peeled coating	F1	9.99%	0.2779	0.2497	0.3037

Estimates are equal-weight means across nine models using canonical majority-voted predictions. Precision, recall, and F1 were computed within each model before averaging. Intervals use 1,000 shared image-level bootstrap resamples (seed 20260823) and the 2.5th and 97.5th percentiles. Zero-denominator values were set to zero. Estimates and interval limits are on the 0-1 scale; prevalence is a percentage. Intervals are exploratory and do not account for within-participant dependence.

Supplementary Table S8. Canonical thin-tongue and thin-coating audit under KGRP.

Model	Predicted thin tongue	Predicted thin coating	Predicted thin tongue and reference thin coating	Predicted thin coating and reference thin tongue
Doubao-Seed-2.0-Pro	119	1867	86	368
Gemini-3.1-Pro	12	1697	9	343
Claude-Sonnet-4.6	0	1729	0	314
Kimi-K2.6	21	1973	16	392
Qwen3.6-Plus	2	1988	1	389
GPT-5.3	0	2037	0	387
GLM-4.6V	0	2105	0	416
Qwen3-Omni-30B-A3B	18	2282	15	430
MiniMax-M2.7	0	12	0	2

Counts use canonical majority-voted predictions on 2,694 images. The final two columns describe co-occurrence with the reference label, not proven substitution errors: thin tongue and thin coating belong to different ontology domains and may coexist. They should not be interpreted as a confusion matrix.

Supplementary Table S9. Canonical attempt-level and majority-voted performance.

Strategy	Micro-F1	Macro-F1	Sample Jaccard	Hamming loss	Exact match
Attempt 1	47.39%	33.47%	34.16%	26.70%	0.41%
Attempt 2	47.17%	33.25%	33.88%	26.65%	0.43%
Attempt 3	47.20%	33.33%	33.91%	26.67%	0.41%
Majority vote	47.92%	33.07%	34.75%	26.04%	0.43%

Values are equal-weight means over the 27 model-prompt conditions in the full benchmark. Attempt-level metrics use the available records for each attempt index, so image counts can differ when generations are missing. Majority-voted metrics use 2,694 images per condition. This is a descriptive summary, not a paired test of the effect of voting.

Supplementary Table S10. Cross-model means, medians, and interquartile ranges.

Prompt or contrast	Metric	Mean	Median	IQR
DP	Micro-F1	47.57	54.40	49.27 to 55.38
	Macro-F1	32.41	38.07	28.97 to 40.52
	Sample-Jaccard	34.45	39.52	35.08 to 40.25
	Hamming-loss	26.39	27.12	26.07 to 27.81
	Exact-match	0.40	0.45	0.37 to 0.45
KGP	Micro-F1	47.68	53.52	49.72 to 54.84
	Macro-F1	33.22	38.85	29.86 to 40.88
	Sample-Jaccard	34.46	38.59	35.67 to 39.59
	Hamming-loss	26.20	25.98	25.33 to 27.09
	Exact-match	0.40	0.45	0.26 to 0.63
KGRP	Micro-F1	48.51	55.18	49.98 to 55.67
	Macro-F1	33.60	39.24	29.26 to 40.79
	Sample-Jaccard	35.32	40.13	35.75 to 40.45
	Hamming-loss	25.54	24.85	24.00 to 26.79
	Exact-match	0.49	0.45	0.30 to 0.63
KGRP-DP (pp)	Micro-F1	0.94	0.69	0.29 to 2.09
	Macro-F1	1.19	1.42	0.27 to 2.20
	Sample-Jaccard	0.87	0.61	0.15 to 2.16
	Hamming-loss	-0.85	-1.00	-1.71 to -0.01
	Exact-match	0.09	0.00	-0.04 to 0.19

Metric levels are percentages; KGRP-minus-DP changes are percentage points. Summaries describe the nine fixed models on 2,694 images. Quartiles use linear interpolation. Paired-change medians are calculated from the nine model-specific changes, not by subtracting prompt-wise medians. IQRs describe model variation and are not confidence intervals.

Supplementary Table S11. KGRP-minus-DP differences with 95% image-bootstrap percentile intervals.

Model	Micro-F1	Macro-F1	Sample Jaccard	Hamming loss	Exact match
Doubao-Seed-2.0-Pro	+2.09 (+1.71, +2.45)	+2.20 (+1.52, +2.90)	+2.16 (+1.76, +2.55)	-3.05 (-3.30, -2.81)	+0.26 (-0.04, +0.56)
Gemini-3.1-Pro	+3.65 (+3.08, +4.21)	+1.60 (+0.90, +2.35)	+3.69 (+3.13, +4.25)	-2.07 (-2.43, -1.70)	+0.59 (+0.26, +0.97)
Claude-Sonnet-4.6	+0.29 (-0.12, +0.68)	+0.27 (-0.28, +0.79)	+0.17 (-0.22, +0.57)	-0.01 (-0.26, +0.28)	+0.00 (-0.19, +0.19)
Kimi-K2.6	+2.35 (+1.76, +2.91)	+2.32 (+1.47, +3.12)	+2.50 (+1.93, +3.05)	-1.71 (-2.04, -1.38)	+0.19 (-0.19, +0.56)
Qwen3.6-Plus	+0.69 (+0.21, +1.16)	-0.41 (-1.29, +0.40)	+0.61 (+0.16, +1.11)	-1.00 (-1.30, -0.69)	+0.07 (-0.22, +0.37)
GPT-5.3	-1.11 (-1.53, -0.66)	+1.42 (+0.76, +2.06)	-1.27 (-1.69, -0.85)	+1.74 (+1.50, +1.95)	-0.07 (-0.41, +0.26)
GLM-4.6V	+1.32 (+0.83, +1.84)	+2.83 (+2.09, +3.53)	+0.94 (+0.45, +1.43)	-0.36 (-0.62, -0.10)	-0.22 (-0.52, +0.07)
Qwen3-Omni-30B-A3B	-1.19 (-1.79, -0.56)	+0.30 (-0.56, +1.16)	-1.16 (-1.72, -0.58)	-1.17 (-1.51, -0.85)	-0.04 (-0.37, +0.30)
MiniMax-M2.7	+0.36 (+0.18, +0.58)	+0.18 (+0.08, +0.29)	+0.15 (+0.07, +0.25)	-0.01 (-0.04, +0.01)	+0.00 (+0.00, +0.00)
Fixed nine-model mean	+0.94 (+0.77, +1.10)	+1.19 (+0.93, +1.44)	+0.87 (+0.71, +1.02)	-0.85 (-0.94, -0.76)	+0.09 (-0.02, +0.19)

Values are percentage-point differences (95% percentile interval) from canonical voted predictions on 2,694 images. The 1,000 bootstrap resamples (seed 20260823) use identical image indices across models and prompts. The aggregate estimand is the equal-weight mean of within-model contrasts across nine fixed models. Positive differences favor KGRP except for Hamming loss. Intervals assume image independence, are not multiplicity corrected, and do not establish patient-level or model-population superiority.

Supplementary Table S12A. Raw-output audit denominators for all 27 model-prompt conditions.

Model	Prompt	Recorded	Missing	Parse failures	Parsed	Valid empty
Qwen3-Omni-30B-A3B	DP	8082	0	17	8065	94
	KGP	8082	0	22	8060	94
	KGRP	8082	0	30	8052	164
Claude-Sonnet-4.6	DP	8082	0	5	8077	0
	KGP	8082	0	10	8072	0
	KGRP	8082	0	7	8075	0
MiniMax-M2.7	DP	8082	0	7369	713	695
	KGP	8082	0	7584	498	429
	KGRP	8082	0	7214	868	476
Doubao-Seed-2.0-Pro	DP	8082	0	0	8082	6
	KGP	8082	0	0	8082	4
	KGRP	8082	0	0	8082	2
Gemini-3.1-Pro	DP	8082	0	0	8082	1
	KGP	8082	0	0	8082	1
	KGRP	8082	0	0	8082	1
Kimi-K2.6	DP	8082	0	58	8024	599
	KGP	8082	0	81	8001	561
	KGRP	8082	0	89	7993	9
GPT-5.3	DP	8082	0	0	8082	2
	KGP	8082	0	0	8082	7
	KGRP	8082	0	0	8082	7
Qwen3.6-Plus	DP	8077	5	11	8066	7
	KGP	8077	5	5	8072	4
	KGRP	8075	7	6	8069	3
GLM-4.6V	DP	8022	60	33	7989	3
	KGP	7728	354	25	7703	3
	KGRP	7736	346	58	7678	9

Each condition has 8,082 planned generation slots (2,694 images x 3 requested generations). Recorded plus missing equals planned; parse failures plus parsed equals recorded. Parsed generations include valid empty normalized label sets. Original raw parse flags are preserved. Canonical record counts and parse classifications may differ; absent records do not establish whether an API request was executed.

Supplementary Table S12B. Raw and post-processed ontology violations.

Model	Prompt	Raw conflict / parsed	Raw incidence %	Raw rule count	Raw E_single %	Post counts generations / rules
Qwen3-Omni-30B-A3B	DP	28/8065	0.347	44	0.045	0 / 0
	KGP	88/8060	1.092	112	0.116	0 / 0
	KGRP	30/8052	0.373	39	0.040	0 / 0
Claude-Sonnet-4.6	DP	73/8077	0.904	73	0.075	0 / 0
	KGP	106/8072	1.313	106	0.109	0 / 0
	KGRP	0/8075	0.000	0	0.000	0 / 0
MiniMax-M2.7	DP	0/713	0.000	0	0.000	0 / 0
	KGP	1/498	0.201	1	0.017	0 / 0
	KGRP	0/868	0.000	0	0.000	0 / 0
Doubao-Seed-2.0-Pro	DP	45/8082	0.557	45	0.046	0 / 0
	KGP	82/8082	1.015	82	0.085	0 / 0
	KGRP	0/8082	0.000	0	0.000	0 / 0
Gemini-3.1-Pro	DP	15/8082	0.186	15	0.015	0 / 0
	KGP	52/8082	0.643	55	0.057	0 / 0
	KGRP	0/8082	0.000	0	0.000	0 / 0
Kimi-K2.6	DP	57/8024	0.710	63	0.065	0 / 0
	KGP	60/8001	0.750	65	0.068	0 / 0
	KGRP	0/7993	0.000	0	0.000	0 / 0
GPT-5.3	DP	10/8082	0.124	10	0.010	0 / 0
	KGP	17/8082	0.210	20	0.021	0 / 0
	KGRP	3/8082	0.037	3	0.003	0 / 0
Qwen3.6-Plus	DP	32/8066	0.397	33	0.034	0 / 0
	KGP	51/8072	0.632	54	0.056	0 / 0
	KGRP	8/8069	0.099	9	0.009	0 / 0
GLM-4.6V	DP	17/7989	0.213	24	0.025	0 / 0
	KGP	67/7703	0.870	91	0.098	0 / 0
	KGRP	9/7678	0.117	9	0.010	0 / 0

Raw incidence counts parsed generations with at least one violation. Raw E_{single} divides violated rule checks by 12 times parsed generations. Applying the same 12 rules to the same eligible generations yields zero post-processing conflict-containing generations and violated rules in each condition; corresponding rates are also zero. These zeros measure deterministic rule enforcement, not intrinsic model consistency.

Pooled raw conflict incidence across models was DP: 277/65,180 (0.425%); KGP: 524/64,652 (0.810%); KGRP: 50/64,981 (0.077%). Manuscript Table 3 instead reports equal-weight means of model-specific rates. Across all conditions, 851 of 194,813 parsed generations had a conflict (0.437%), with 953 violated rule checks, 22,624 parse failures, 3,181 valid empty normalized outputs, and 777 missing generation slots.

Supplementary Table S13. Conflict resolution sensitivity

We reanalyzed the retained generations under four evaluator policies: the archived benchmark-frequency rule; a fixed priority based on the existing 22-label ontology order; frequencies computed only from the 2,154 supervised training images; and no mutual-exclusion removal. The fixed order was selected without inspecting its scores and uses no reference-label frequencies. It is a deterministic sensitivity comparator, not a validated clinical priority. Rules were applied in the same sequential 12-pair order at attempt level. All policies retained the same image identifiers, canonical parsing flags, alias mapping, usable-label conventions, retained-attempt counts, strict-majority rule, and metric definitions. No new model inference or training was performed.

Before comparing policies, we reconstructed the benchmark-frequency pipeline and verified exact equality with all 27 archived canonical voted prediction matrices. We then recomputed Micro-F1, Macro-F1, sample-level Jaccard, Hamming loss, and exact-match ratio under each policy, reporting model-specific scores and equal-weight nine-model means on both the full 2,694-image set and the same 270 test images. Training-frequency priorities excluded validation and test images, but only the 270-image test analysis is held out from that frequency estimate; the full-set analysis includes the training images. Comparisons are descriptive sensitivity analyses without additional superiority tests. Supplementary Table S13 reports the priorities, aggregate scores, prompt contrasts, and prediction changes; the accompanying XLSX provides all model-specific results.

S13A. Equal-weight means across nine models on the full 2,694-image benchmark. Values are percentages.

Policy	Prompt	Micro-F1	Macro-F1	Jaccard	Hamming loss	Exact match
B	DP	47.570	32.407	34.455	26.387	0.400
T	DP	47.570	32.407	34.455	26.387	0.400
F	DP	47.574	32.491	34.463	26.382	0.400
U	DP	47.575	32.499	34.462	26.389	0.400
B	KGP	47.676	33.217	34.465	26.197	0.404

T	KGP	47.676	33.217	34.465	26.197	0.404
F	KGP	47.685	33.397	34.479	26.186	0.408
U	KGP	47.692	33.405	34.483	26.197	0.404
B	KGRP	48.508	33.597	35.321	25.537	0.487
T	KGRP	48.508	33.597	35.321	25.537	0.487
F	KGRP	48.504	33.592	35.317	25.537	0.487
U	KGRP	48.507	33.595	35.320	25.538	0.487

B = benchmark frequency; T = training frequency; F = fixed ontology order; U = no conflict removal.

S13B. KGRP-minus-DP mean contrasts.

Scope	Policy	Micro-F1	Macro-F1	Jaccard	Hamming loss	Exact match
Full	B	+0.939	+1.190	+0.866	-0.850	+0.087
Test	B	+0.834	+0.875	+0.777	-0.666	+0.247
Full	T	+0.939	+1.190	+0.866	-0.850	+0.087
Test	T	+0.834	+0.875	+0.777	-0.666	+0.247
Full	F	+0.930	+1.100	+0.854	-0.845	+0.087
Test	F	+0.832	+0.907	+0.778	-0.660	+0.247
Full	U	+0.932	+1.096	+0.858	-0.851	+0.087
Test	U	+0.834	+0.875	+0.778	-0.668	+0.247

Values are percentage points. Positive values favor KGRP except for Hamming loss, for which negative values are favorable. Full = 2,694 images; test = the 270-image subset held out from training-frequency estimation. No interval or superiority test is implied.

S13C. Rule-level priorities.

Rule pair	B support A/B	T support A/B	B and T retain	F retains
Tender / Tough	362/472	281/373	Tough	Tender
Enlarged / Thin tongue	625/506	509/386	Enlarged	Enlarged
Thin coating / Thick coating	1812/760	1446/610	Thin coating	Thin coating
No coating / Thin coating	101/1812	80/1446	Thin coating	No coating
No coating / Thick coating	101/760	80/610	Thick coating	No coating
No coating / Moist	101/317	80/269	Moist	No coating
No coating / Dry	101/537	80/428	Dry	No coating
No coating / Rotten/greasy	101/421	80/341	Rotten/greasy	No coating
No coating / Peeled	101/269	80/213	Peeled	No coating
No coating / White	101/1780	80/1428	White	No coating
No coating / Yellow	101/619	80/489	Yellow	No coating
No coating / Gray-black	101/576	80/464	Gray-black	No coating

Frequencies were counted from the full 2,695-record label workbook for B and the 2,154 training images for T. The extra workbook record contributes no positive label counts. The fixed ontology order was the existing column order, with the earlier label retained. Rule application follows the row order below. Frequency ties retain the lexically earlier canonical Chinese label; no tie occurred for these 12 pairs.

Training and benchmark frequencies yielded identical priorities and predictions in every condition. Relative to B, F changed voted predictions in 206 image-model-prompt combinations (286 binary label decisions), and U changed 185 combinations (185 decisions), among 72,738 full-set combinations. On the test subset, these counts were 20 combinations (31 decisions) and 16 combinations (16 decisions), respectively, among 7,290 combinations. These are condition-specific counts, not unique images or participants. The accompanying sensitivity analysis XLSX provides all 216 model-prompt-policy-scope rows and the source provenance.

Supplementary Table S14. Retained generation counts

Model	Prompt	G0	G1	G2	G3	Recorded	Missing	Cache
Doubao-Seed-2.0-Pro	DP	0	0	0	2694	8082	0	0
Doubao-Seed-2.0-Pro	KGP	0	0	0	2694	8082	0	0
Doubao-Seed-2.0-Pro	KGRP	0	0	0	2694	8082	0	0
Qwen3-Omni-30B-A3B	DP	0	0	0	2694	8082	0	0
Qwen3-Omni-30B-A3B	KGP	0	0	0	2694	8082	0	0
Qwen3-Omni-30B-A3B	KGRP	0	0	0	2694	8082	0	0
Kimi-K2.6	DP	0	0	0	2694	8082	0	0
Kimi-K2.6	KGP	0	0	0	2694	8082	0	0
Kimi-K2.6	KGRP	0	0	0	2694	8082	0	0
MiniMax-M2.7	DP	0	0	0	2694	8082	0	0
MiniMax-M2.7	KGP	0	0	0	2694	8082	0	0
MiniMax-M2.7	KGRP	0	0	0	2694	8082	0	0
Qwen3.6-Plus	DP	1	1	0	2692	8077	5	1
Qwen3.6-Plus	KGP	1	1	0	2692	8077	5	1
Qwen3.6-Plus	KGRP	1	1	2	2690	8075	7	1
GLM-4.6V	DP	0	30	0	2664	8022	60	0
GLM-4.6V	KGP	0	177	0	2517	7728	354	0
GLM-4.6V	KGRP	0	173	0	2521	7736	346	0
Gemini-3.1-Pro	DP	0	0	0	2694	8082	0	0
Gemini-3.1-Pro	KGP	0	0	0	2694	8082	0	0
Gemini-3.1-Pro	KGRP	0	0	0	2694	8082	0	0
Claude-Sonnet-4.6	DP	0	0	0	2694	8082	0	0
Claude-Sonnet-4.6	KGP	0	0	0	2694	8082	0	0
Claude-Sonnet-4.6	KGRP	0	0	0	2694	8082	0	0
GPT-5.3	DP	0	0	0	2694	8082	0	0
GPT-5.3	KGP	0	0	0	2694	8082	0	0
GPT-5.3	KGRP	0	0	0	2694	8082	0	0

G0–G3 count image-model-prompt combinations with 0–3 retained generation records. Each condition has 2,694 images and 8,082 planned slots. Recorded = G1 + 2G2 + 3G3; missing = 8,082 - recorded. The cache column counts zero-generation records whose cached empty set supplies a scoring vector. Scored vectors = recorded + cache. Parsing success does not define G_i or K_i. Raw failures and valid empty parsed outputs remain separately reported in S12.

Across all conditions: G0 = 3, G1 = 383, G2 = 2, G3 = 72,350; recorded = 217,437 and missing = 777. The three cached empty vectors all belong to Qwen3.6-Plus, image Tongue-5375-0761, one per prompt. Thus, total scored vectors = 217,440 and the Ki distribution is K0 = 0, K1 = 386, K2 = 2, K3 = 72,350. These cached sets are not counted as generated or successfully parsed responses. The 72,738 combinations reuse the same 2,694 images and are not independent participants.
